# Qluster: An easy-to-implement generic workflow for robust clustering of health data

**DOI:** 10.3389/frai.2022.1055294

**Published:** 2023-02-06

**Authors:** Cyril Esnault, Melissa Rollot, Pauline Guilmin, Jean-Daniel Zucker

**Affiliations:** ^1^Quinten, Paris, France; ^2^Sorbonne University, IRD, UMMISCO, Bondy, France; ^3^Sorbonne University, INSERM, NUTRIOMICS, Paris, France

**Keywords:** clustering, easy-to-implement algorithm, robustness, health data, genericity

## Abstract

The exploration of heath data by clustering algorithms allows to better describe the populations of interest by seeking the sub-profiles that compose it. This therefore reinforces medical knowledge, whether it is about a disease or a targeted population in real life. Nevertheless, contrary to the so-called conventional biostatistical methods where numerous guidelines exist, the standardization of data science approaches in clinical research remains a little discussed subject. This results in a significant variability in the execution of data science projects, whether in terms of algorithms used, reliability and credibility of the designed approach. Taking the path of parsimonious and judicious choice of both algorithms and implementations at each stage, this article proposes Qluster, a practical workflow for performing clustering tasks. Indeed, this workflow makes a compromise between (1) genericity of applications (e.g. usable on small or big data, on continuous, categorical or mixed variables, on database of high-dimensionality or not), (2) ease of implementation (need for few packages, few algorithms, few parameters, ...), and (3) robustness (e.g. use of proven algorithms and robust packages, evaluation of the stability of clusters, management of noise and multicollinearity). This workflow can be easily automated and/or routinely applied on a wide range of clustering projects. It can be useful both for data scientists with little experience in the field to make data clustering easier and more robust, and for more experienced data scientists who are looking for a straightforward and reliable solution to routinely perform preliminary data mining. A synthesis of the literature on data clustering as well as the scientific rationale supporting the proposed workflow is also provided. Finally, a detailed application of the workflow on a concrete use case is provided, along with a practical discussion for data scientists. An implementation on the Dataiku platform is available upon request to the authors.

## 1. Introduction

Health data are of great importance to public health, research, and medical development. It is any data related to the health conditions, outcomes, and quality of life of an individual or population. Health data may be collected during the course of ongoing patient care (e.g., claims data, medical records, administrative data) or as part of a formal clinical trial program.

In health data analysis, clustering methods are a primary tool, for finding pockets of homogeneity within a heterogeneous population, to uncover different disease phenotypes, stages of a disease, or variations in disease outcomes (Fränti et al., 2022). A precise understanding of the clusters of patients suffering from a disease ultimately allows for the overall improvement of their care (Windgassen et al., 2018). In this respect, there is extensive literature to discuss clustering tasks, be it for the choice of appropriate clustering methods (Obembe and Oyelade, 2019), for the

clustering algorithms for large data (Shirkhorshidi et al., 2014), for clustering methods for qualitative/mixed data (Hennig and Liao, 2013), for methods to assess clustering quality, stability, and the number of clusters (Lange et al., 2004; Nietto and Maria, 2017), or for performing in-depth comparative statistical analysis of methods (Jain, 2010; Nagpal et al., 2013).

The profusion of methods makes it difficult for most data scientists to choose and systematically apply a methodology that is complete, reasonably fast, and satisfactory from a robustness point of view with respect to the clinical question they are trying to answer. The data scientist is indeed confronted with a very wide range of choices regarding both the algorithms and their implementations (including R and Python), in particular, according to the nature of the data and their volume. Furthermore, contrary to the more “conventional” bio-statistical methods in clinical studies, the lack of clear guidelines on which data science approaches to use leads to a greater subjectivity in the choice of approaches, and in particular, those of clustering in clinical data. This makes the statistical analysis plans for observational studies proposed by data scientists and the results obtained more variable.

The clustering process involves many decision steps, from the data preparation step to the evaluation of clusters' stability and clusters' description. To the authors' knowledge, there is not yet a single, simplified workflow in the literature that is easy to implement for both expert and non-expert health data scientists (with off-the-shelf tools in R or Python), well-supported by the literature, generic (e.g., regardless of the nature -continuous/binary/categorical/mixed- or volume of data -small/large), which facilitates its routine application. Most of the articles that come close to this goal focus on process automation (autoML, e.g., Kamoshida and Fuyuki, 2020) or clustering methods comparison (Wiwie et al., 2015). This study (obviously) does not pretend to impose a single solution to a clustering problem as experience and literature have both shown that there is no single solution to a clustering task [refer to in particular Kleinberg (2002)]. However, this study aims to give to give data scientists guidance through an easy framework that can be used in routine practice in a wide variety of cases. This article is, thus, intended to:

i health data scientists, companies, or institutions that need a general workflow for routine—possibly automated—clustering projects on data of various types and volumes (e.g., for preliminary data mining), or
ii health data scientists with limited experience, who are looking for both an overview of the literature and an accessible and reusable workflow with concrete practical recommendations to quickly implement a complete unsupervised clustering approach adapted to various projects.

In this article, we propose in Section 2, a synthesis of the different methods related to unsupervised clustering in the literature and in relation to the implementations available in R or Python. In Section 3, we propose *Qluster*, a generic workflow for clustering tabular data of any nature and size, while considering *(1)* the literature guidelines on how to perform robust clustering, and *(2)* the availability and ease of use of R or Python implementations for data scientist users. Then, in Section 4, we detail this workflow through a step-by-step application on the open-access Cardiovascular Disease^1^ practice dataset to help data scientists to reapply this workflow on their own project with concrete recommendations. We then provide a practical discussion in Section 5 and conclude in Section 6. Data scientists will also find in both Sections 2 and 4 all the necessary literature (rationale) to support the use of this workflow, which greatly facilitates the tedious work of writing the statistical analysis plan with innovative approaches (data science) in clinical research.

## 2. Statistical rationale and literature review on data clustering

**Figure 1:**
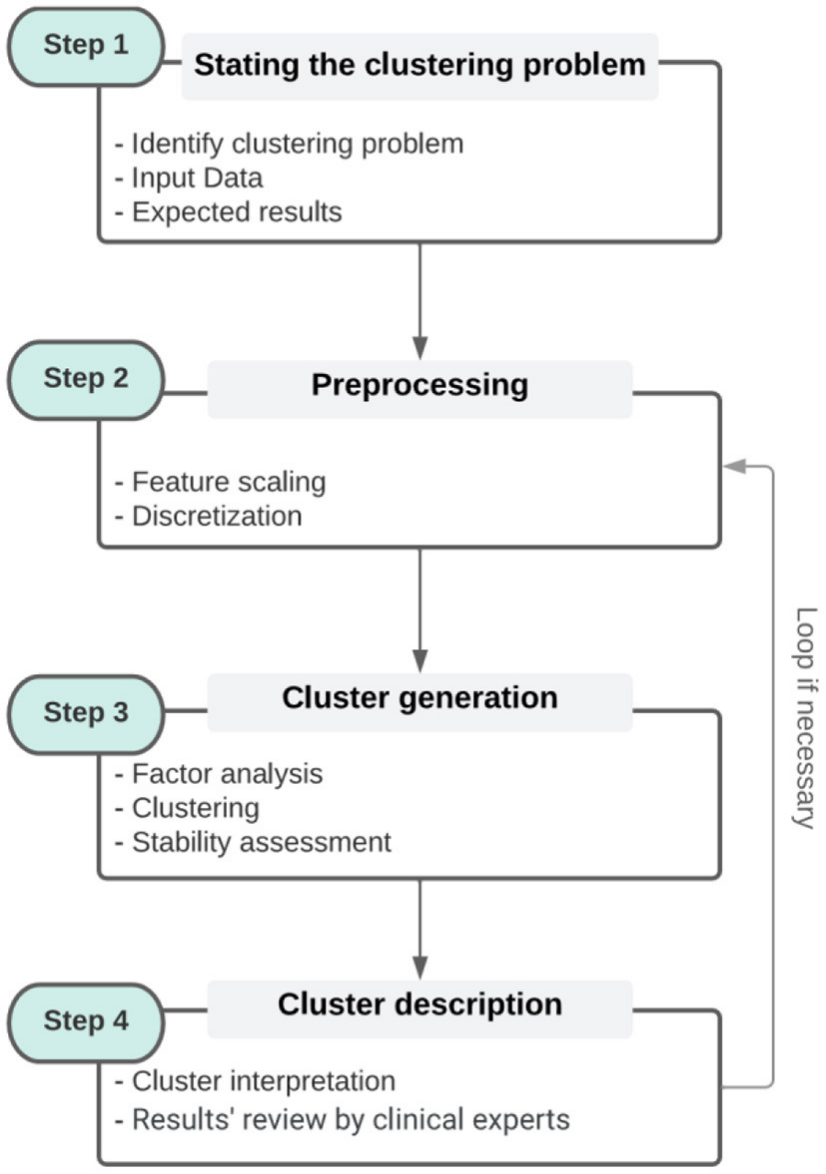
The general clustering process [It is broken down into four steps. Step 1 corresponds to the identification of the problem and the collection of data. Step 2 is a pre-processing step that includes the different transformations of the data. Step 3 is the clustering of the data itself, while step 4 is the interpretation of the clusters with respect to the original data. The contents of each box (steps) are examples and not sub-steps that must be followed].

This section discusses the state-of-the-art of clustering methods in the general clustering process (refer to Figure 1, steps 2–4. Step 2 will be discussed both in this section and in Section 4 through an illustrative example).

### 2.1. Overview of unsupervised clustering methods

As generally defined, clustering is “the task of grouping a set of objects in such a way that objects in the same group (called a cluster) are more similar (in some sense) to each other than to those in other groups (clusters)” (Altman and Krzywinski, 2017). Such a task is used to group homogeneous subsets of observations to better understand their global heterogeneity. This is, particularly, true in clinical data analysis to describe disease heterogeneity, stratify patients, and obtain profiles of targeted populations. The result of a clustering task is in general an assignment of the input data into a fixed number of clusters. Two categories of clustering methods are usually distinguished according to the nature of such assignment: hard and soft clustering methods. Hard clustering provides a partition in which each object in the data set is assigned to one and only one cluster. Soft (or Fuzzy) clustering generates a fuzzy partition that provides a degree of membership of each object to a given cluster. This gives the possibility to express that objects belong to more than one cluster at the same time. It is of note that the definition of a cluster itself is not very precise, which partly explains why there are so many clustering algorithms (Estivill-Castro, 2002).

**Figure 2:**
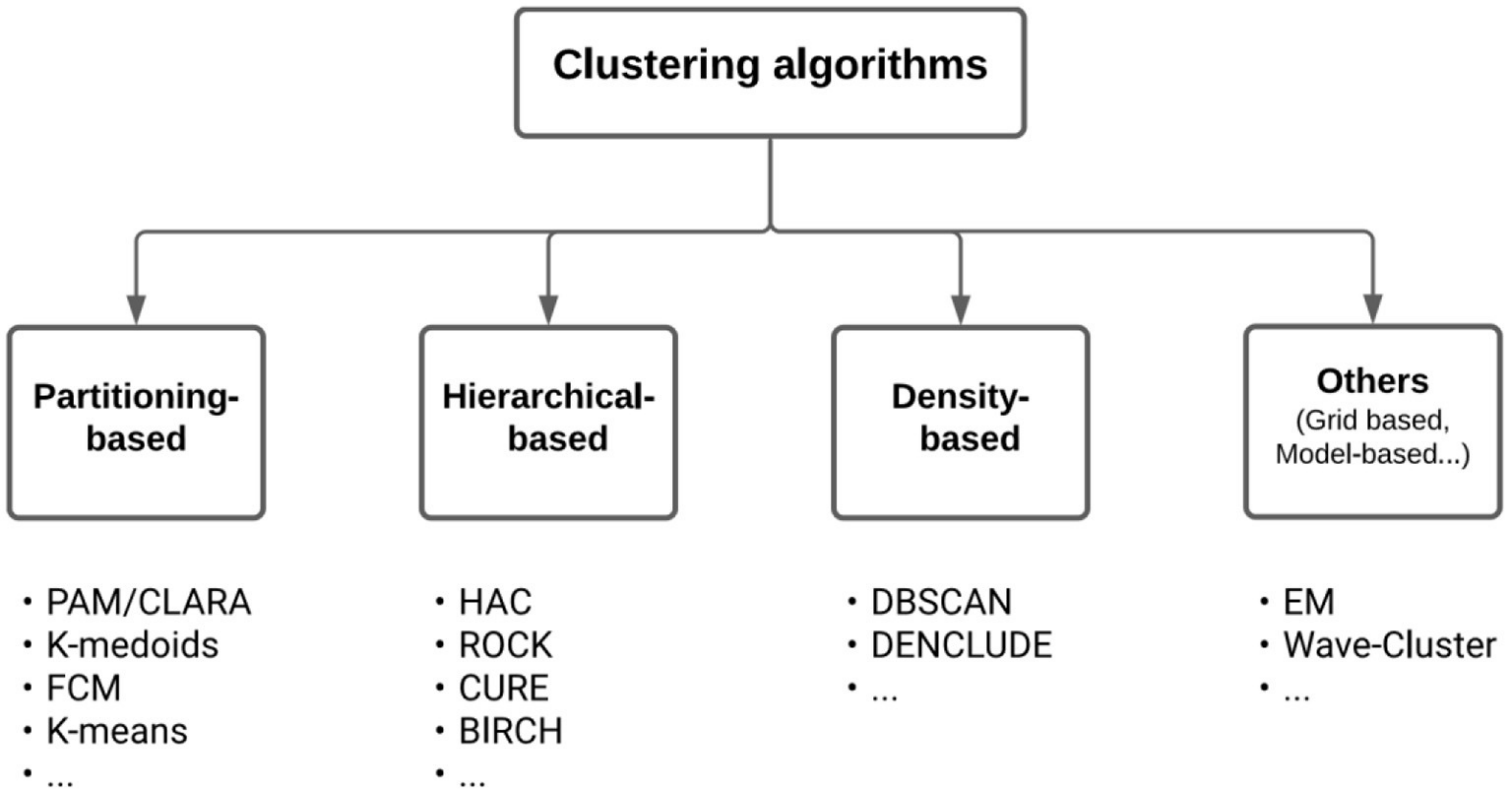
A taxonomy of clustering algorithms. Below the boxes are listed well-known algorithms of the corresponding types.

In the field of Machine Learning, clustering methods pertain to the so-called unsupervised learning methods. Clustering should not be confused with the field of Subgroup Discovery, which also aims at finding groups but in a supervised way, for example, to identify prognostic factors of an outcome or predictive factors of the treatment effect on an outcome (Zhou et al., 2019; Esnault et al., 2020). The many clustering algorithms that exist in the literature (Xu and Donald, 2010; Fahad et al., 2014; Ahmad and Khan, 2019) can be classified according to the cluster models (e.g., centroid, connectivity, distribution, density, and graph). Among the wide variety of methods, they are three main types, all producing a hard partition of the observations (Figure 2, which is adapted from Figure 1 in Fahad et al., 2014):

- **Partitioning-based methods**^2^ [e.g., *K*-means (MacQueen, 1967), *K*-medoid (Jin and Jiawei, 2010), PAM (Ng and Han, 1994), *K*-modes (Huang, 1997), *K*-prototype (Huang, 1998), CLARA (Kaufman and Rousseeuw, 2009), and FCM (Bezdek et al., 1984)].
- **Hierarchical-based methods** [e.g., BIRCH (Zhang et al., 1996), CURE (Guha et al., 1998), and ROCK (Guha et al., 2000)].
- **Density-based methods** [e.g., DBSCAN (Ester et al., 1996) and DENCLUDE (Hinneburg and Keim, 1998)].

The **first** type of method is considered to be the most popular class of clustering algorithms for its ease-of-implementation, simplicity, efficiency, and empirical success (Jain, 2010). It aims at directly obtaining a single data partition into *K* clusters. Partitioning-based methods require setting the number *K* of clusters, which are rarely known *a priori* but can be estimated from the data using several known methods (Caliński and Harabasz, 1974; Milligan and Martha, 1985; Gordon, 1999; Halkidi et al., 2001; Meilă, 2007; Hennig and Liao, 2013; Hennig, 2014). These include the optimization of internal validity metrics that reflect the compactness and separation of the clusters (e.g., Average Silhouette Width, Davies–Bouldin index, Calinski–Harabasz index, and Dunn index). Equally, some of the partitioning-based methods rely on random initialization of different *K*-centroids that can lead to different outputs (local optimum), non-reproducible clusters, or wrong or empty clustering. Some solutions exist, such as the *K*-means++ algorithm, which includes a smart centroid initialization method for the *K*-means algorithm (Arthur and Vassilvitskii, 2007). The goal is to spread out the initial centroid by assigning the first centroid randomly and then selecting the rest of the centroids based on the maximum squared distance. The idea is, therefore, to push the centroids as far as possible from one another. Similarly, the PAM algorithm is a deterministic *K*-medoid algorithm that directly integrates an initialization procedure called BUILD. During the BUILD phase, the first medoid is selected to be the one that has the minimum cost, with cost being the sum over all distances to all other points.

The **second** type produces a hierarchy of clusters, called a dendrogram, especially useful when one needs several hard partitions at different hierarchical levels (i.e., from a macro vision with a few groups to a micro vision with many groups). These hierarchical methods have a major drawback though, once a step (merge or split) is performed, it is not undone, potentially making erroneous decisions impossible to correct: they are often greedy algorithms that optimize local criteria without backtracking, whereas the clustering problem is (by definition) a global optimization problem. Moreover, hierarchical-based methods generally have higher time and space complexities than partitioning-based methods and rely on more input parameters that leave more room for subjectivity regarding the choice of settings, with a direct impact on the generated clusters (Fahad et al., 2014). Some studies have also shown that hierarchical-based algorithms lead to worse clustering results than partitioning algorithms, suggesting that the latter are well-suited for clustering large datasets due to not only their relatively low computational requirements but also comparable or even better clustering performance (Zhao and Karypis, 2002; Kaushik and Bhawana, 2014).

The **third** type of method does not explicitly require a number of clusters, nor does it rely on a distance threshold from a “center” (like partitioning-based methods do). On the contrary, density-based methods rely on the estimated density of observations to perform the partitioning. Such a method is, in this sense, more local and allows us to represent clusters whose topology is less induced by the sole distance used (like hyper-spheres when using the Euclidean distance in partitioning-based methods). This strategy may, however, be associated with a greater propensity to overfit data and greater difficulties to set up the hyper-parameters.

Beyond these three main types of methods, there are many other alternative clustering approaches including grid-based methods [e.g., Wave-Cluster (Sheikholeslami et al., 1998) and STING (Wang et al., 1997)]. They perform the clustering on grids rather than on the whole dataset. There are also model-based methods that optimize the fit between the data and predefined models, assuming that the data are generated by a mixture of underlying probability distribution e.g., mixture density model [EM (Do and Serafim, 2008)], conceptual clustering [COBWEB (Fisher, 1987)], and neural networks model [SOMs (Ciampi and Yves, 2000)]. More recently, a wide range of new approaches to clustering based on deep learning have emerged (Aljalbout et al., 2018); they are mainly used to cluster unstructured data. Deep neural networks (DNN) can be effective ways to map a high-dimensional data space to a lower-dimensional feature space. Nevertheless, DNN often requires large datasets and a procedure for *post-hoc* interpretability of the clusters (the representation learned by DNN architectures is not easily understandable). Finally, recent methods for graph clustering focus more specifically on finding sets of nodes in networks or graphs that have more connections within the set than outside the set (Sieranoja and Pasi, 2022). For more information on the types of clustering algorithms and their suitability to the data types (categorical, text, multimedia, stream, and time series), refer to Oyelade et al. (2019).

### 2.2. Choosing an appropriate clustering approach

The choice of the appropriate approach to be used relies on many aspects (Fahad et al., 2014; Ahmad and Khan, 2019) such as:

i its ability to handle the desired type of data (binary/nominal/ordinal/numerical),
ii the dimensionality of the data (refer to e.g., Mittal et al., 2019),
iii the size of the data (small to large data),
iv the availability of reliable implementations in software (e.g., R and Python—the two most popular statistical softwares for data scientists).

Partitioning-based methods are known to be composed of many variants to directly handle continuous (*K*-means, PAM, CLARA, and FCM), categorical (*K*-modes and *K*-medoid), and mixed variables [*K*-prototype and KAMILA (Foss and Marianthi, 2018)]. In addition, the ability of some of these algorithms to directly handle input dissimilarity matrices facilitates the pre-transformation of the original data into data of the desired type prior to clustering, using suited distance measures (e.g., McCane and Michael, 2008). This strategy is, particularly, used to convert categorical or mixed data into numerical data, as there is more literature and algorithms implemented in software for continuous data (e.g., scikit-learn^3^ in Python, or *cluster*^4^ and *FPC*^5^ R packages. The two latter packages provide both a large number of clustering and cluster stability assessment methods and functions to compute dissimilarity matrices and describe the results). Another known alternative consists of one-hot-encoding categorical data into binary variables and treating the latter as continuous (e.g., Li and Latecki, 2017). It is, however, necessary to down-weight the variables obtained, so that no more weight is given to the original variables with more modalities. Finally, dimensionality reduction methods, such as factor analysis [Principal Component Analysis (PCA) for continuous data, Multiple Correspondence Analysis (MCA) for qualitative data, and Factor Analysis of Mixed Data (FAMD) for mixed data (Pagès and Husson, 2017)], can be used before the clustering as a first step to transform the data into numerical components (i.e., the coordinates of the observations on each dimension).

Factor analysis has many other advantages for clustering tasks, such as reducing the dimensionality (making easier the clustering task), reducing noises (by removing the last components that only bear random noise, leading to a more robust unsupervised learning), and dealing with variables that carry similar information and/or are highly correlated (Pagès and Husson, 2017). In the case of qualitative data, a convenient practice for accommodating cluster-level observation heterogeneity in MCA is to adopt a two-step sequential, tandem approach (Arabie and Hubert, 1994): in the first step, a low-dimensional representation of the categorical variables is obtained *via* MCA; in the second step, some variety of cluster analyses are used to identify a set of relatively homogeneous observations groups on the basis of the low-dimensional data. In addition to the ease with which the two-step sequential approach can be implemented, there can be substantive reasons for adopting this approach as well (Green and Abba, 1995). Alternative methods consist of using simultaneously both MCA and a clustering approach in a single framework so that the low-dimensional data can be chosen to facilitate the identification of clusters (Bock, 1987; DeSarbo et al., 1991; De Soete and Carroll, 1994). However, these methods lack implementations (both in R and Python), hindering their use within a clustering workflow. Finally, the selection of the number of components to be kept is the key step. This can be based on several methods including permutation tests (Takane and Hwang, 2002), cross-validation-based methods (Bro et al., 2008; Josse et al., 2012), or methods based on the amount of information carried by each of the dimensions, either compared to an average value equivalent to the Kaiser's rule in PCA (Lorenzo-Seva, 2011) or represented by a scree plot (Clausen, 1998; Drennan, 2010). The latter method has been found to perform fairly well and is the most commonly used to select the optimal number of dimensions (Zwick and Wayne, 1986; Bandalos and Boehm-Kaufman, 2010). It consists of looking at the bend in the falling curve (so-called “elbow”) indicating an optimal dimensionality (if there is no obvious elbow, one can choose the number of components just before a flat appears). It has been adapted from PCA (Cattell, 1996) and used in the context of correspondence analysis (Costa et al., 2013). All factor analysis methods can notably be found in R in the well-known *FactoMineR*^6^ package, and in Python in the *prince*^7^ GitHub library (although issues are still open for the latter). Methods for estimating the number of dimensions to be kept can be found in R in many packages such as the *FactoMineR* and *missMDA*^8^, using cross-validation methods, or in the *factoextra*^9^ package (e.g., scree plot). In Python and to the best of the authors' knowledge, one would need to code these methods to apply them as no specific functions were found.

Whether a factor analysis is performed as a first step or not, the need to choose a distance measure is critical, as some of them are only appropriate according to the type of data, or are preferred in some cases. Indeed, continuous data require appropriate distance measures to obtain the dissimilarity matrix (e.g., Euclidean and Manhattan distances), while categorical data are widely handled with simple matching methods (e.g., Hamming distance for symmetric measures, which is equivalent to Manhattan distance on binary variables, and Jaccard similarity coefficient for asymmetric measures to favor positive co-occurrences over the negative ones). Methods to handle mixed data can consist of combining the above-mentioned methods, such as the Gower distance (i.e., simple matching methods and Manhattan distance).

Finally, some aspects need careful attention when dealing with large data. Indeed, the candidate algorithm must handle either or both high dimensionality and a massive number of observations (including outliers/noisy data), which makes difficult, and sometimes impossible, dissimilarity matrices to be computed. Equally, fast running time is essential with large data as the clustering needs to be performed several times, notably to assess cluster stability and optimize the clustering hyperparameters (e.g., in the use case in Section 4, clustering was replicated 550 times). Few strategies exist to deal with massive data, such as relying on algorithms of lower complexity [e.g., *K*-modes and FCM that are O(n), (Fahad et al., 2014)]. The latter, however, is quickly limited as computing time increases linearly with the size of the data. Alternative methods consist of working on approximations (Sieranoja and Pasi, 2019) or subsets of the whole dataset to cluster smaller datasets before generalizing them [e.g., Mini Batch *K*-means (Sculley, 2010), CLARA (Kaufman and Rousseeuw, 2009), and CLARANS (Ng and Jiawei, 2002)].

The CLARA algorithm is an extension to *K*-medoids methods (e.g., PAM), which is known to be more robust than *K*-means-based algorithms as they minimize a sum of dissimilarities instead of a sum of squared Euclidean distances (Jin and Jiawei, 2010). CLARA allows dealing with data containing a large number of observations (more than several thousand) using a sampling approach, in order to reduce computing time and RAM storage problems. Instead of finding medoids for the entire dataset, CLARA considers a small sample of the data and applies the PAM algorithm to generate an optimal set of medoids. CLARA repeats the sampling and clustering processes several times in order to minimize the sampling bias. In practice, its strength lies in the possibility to adjust the number of samples and the sample sizes, in order to make calculation both time acceptable and storage in RAM possible. This is indeed essential to enable the stability of clustering to be assessed and the best partitioning to be found by repeating the clustering process many times. Compared to CLARA, CLARANS presents a trade-off between the cost and the effectiveness of using samples to obtain clustering. Mini-batch *K*-means, CLARA, and CLARANS can all be found both in R (e.g., respectively, *cluster, FPC*, and *QTCAT*^10^ packages) and in Python (e.g., respectively, scikitlearn, *pycluster*^11^, and *pyclustering*^12^). Please note though that both the quality and the maintenance of libraries on GitHub (*QTCAT, pycluster*, and *pyclustering*) cannot be guaranteed by the present authors.

### 2.3. Methods for clusters description

The interpretability of clusters generated by clustering algorithms remains one of the most important challenges in clinical data analysis, as is often the case with machine learning algorithms (Vellido, 2020). Indeed, the best results will only make sense if they are interpretable by end users. Conventional methods do not provide consensus on how to characterize clusters and this is even more valid in the health sector, where the interpretation of clusters is a matter of medical knowledge of the data itself (Kiselev et al., 2019).

The simplest yet most efficient method remains to compute relevant intra- and inter-clusters descriptive statistics using the initial variables to identify a mapping of the generated clusters based on means or median values (resp. proportions) for continuous (resp. categorical) variables. This can be completed by performing clusterwise distributions comparison with overall distributions, as well as using hypothesis testing to identify input variables whose differences are statistically significant between clusters (Bousquet et al., 2015). Such implementations dedicated to clusters' description may be found in R [e.g., the *cluster.varstats()* function in the *FPC* package that also provides tables and plots] and to the best of our knowledge, no such function was found in Python.

Alternative for making the clusters' description step easier may consist of learning an interpretable multiclass supervised classifier (e.g., decision tree) on cluster labels (outcome) to highlight characteristics and specificities associated with each group. Other methods propose to include the interpretability of clusters directly within the clustering algorithm, and not as a step done afterward (Bertsimas et al., 2021), notably by adding tunable parameters related to interpretability (e.g., refer to Saisubramanian et al., 2020, with a Python implementation found on GitHub^13^).

Finally, methods dedicated to the visualization of clusters make it easier for their interpretation, such as PCA, multidimensional scaling (Torgerson, 1952), t-SNE (Van der Maaten and Hinton, 2008), and uniform manifold approximation and projection (UMAP, McInnes et al., 2018).

### 2.4. Methods for clustering validity and stability assessment

The clustering assessment step is an important phase to increase confidence in results and consists of evaluating both the clustering validity and stability.

Regarding the validity of clustering, one can first distinguish the external validity metrics (Rezaei and Pasi, 2016) that can be used to compare the clusters obtained with the ground truth, which is rarely known. Then, the internal validity metrics that assess the goodness of a data partition using quantities inherited from the data, such as compactness (e.g., the maximum pairwise intra-cluster distances), connectedness (e.g., connectivity metric), or separation (Bezdek and Pal, 1998; Handl et al., 2005). The Dunn Validity Index and the Silhouette coefficient are both commonly used metrics, notably to define the optimal number of clusters, as they both assess the separation (i.e., the inter-cluster distances) over the compactness (intra-cluster distances). Although previous works have shown that there is no single internal cluster validation index that outperforms the other indices, Arbelaitz et al. (2013) compared a set of internal cluster validation indices in many distinct scenarios, indicating that the Silhouette coefficient yielded the best results in most cases. Alternatives exist for estimating the number of clusters in a dataset regardless of the clustering methods, such as the “gap statistic” that compares the change in within-cluster dispersion with that expected under an appropriate reference null distribution (Tibshirani et al., 2001).

Regarding the stability of clustering, several methods are proposed in the literature, by repeating the clustering process several times under conditions that are different from those of origin. These include procedures used in bioinformatics that remove one column at a time (Handl et al., 2005; Datta and Somnath, 2006). Several metrics can then be computed between the set of clusters (Brock et al., 2008), such as the average proportion of non-overlap (APN), the average distance (AD), and the figure of merit (FOM). These methods are notably proposed in the *clValid*^14^ R package and its main function *clValid()*. The latter includes many clustering algorithms (*K*-means, DIANA, FANNY, SOMs, SOTA, PAM, CLARA, and AGNES) and allows for a direct assessment of clusters' stability through the “validation” argument.

Other approaches consist in perturbing the original data, either using bootstrapping (Efron, 1979; Efron and Tibshirani, 1994), noising, and/or sampling methods (Hennig, 2008). The Jaccard similarity statistic is then often used as a metric for assessing stability, by computing the similarities of the original clusters to the most similar clusters in the resampled data. Such methods are implemented in the *FPC* R package, notably in the *clusterboot()* function. The latter is an all-inclusive package that also allows for clustering using a wide variety of algorithms (e.g., *K*-means, hierarchical clustering, normal mixture models, PAM, CLARA, DBSCAN, and spectral clustering), making it easy for a data scientist to generate, compare, and assess the stability of clusters.

As for Python, while there are packages to evaluate the internal validity of clusters [Silhouette coefficient, Rand Index, Caliński–Harabasz Index (Caliński and Harabasz, 1974), see in particular the *sklearn.cluster* library], no Python library was found to evaluate the stability of generated clusters. This reinforces the fact that Python does not cover as easily as R the whole clustering process, as there is no Python package that includes all the steps of interest (clusters generation, internal validity evaluation, clustering optimization, clusters stability evaluation, and clusters description), as in the *FPC* R package.

Finally, the methods for testing clustering stability on a hold-out dataset are barely mentioned in the literature. This would consist of pre-allocating the observations in the test set into the clusters obtained from the learning set, and clustering the test dataset to check for good allocation. However, no implementation was found in either R or Python. We can see a simplified application of the clustering of two independent datasets in Saint Pierre et al. (2020).

## 3. The Qluster workflow

### 3.1. Research objective

Many statisticians/data scientists are confronted with a great number of algorithms and implementations for data clustering. This can make it difficult to manage clustering studies and is likely to generate analytical strategies that are insufficiently rigorous, not consensual, or not adapted to the problem. This is, particularly, the case for any statistician/data scientist in contract research organizations that provide support to the healthcare industries, who has the responsibility to conduct clustering analyses but is still little experienced in using them. Our goal is to propose a practical workflow for data scientists because of its **genericity of application** (e.g., usable on small or big data, on continuous, categorical or mixed variables, on database of high-dimensionality or not) while preserving the **simplicity of implementation** and **use** (need for few packages, algorithms, parameters, …) and the **robustness and reliability** of the methodology (e.g., evaluation of the stability of clustering, use of proven algorithms and robust packages, management of noisy and/or collinear data). The objective of this workflow is therefore not to be the solution to all situations but to propose a simple and robust basis that is as generic as possible. In a way, a choice that aims to be “globally optimal” for practice but not optimal in every case. This generic workflow can be useful both for data scientists with little experience in the field to make data clustering easier and more robust, and for more experienced data scientists who are looking for a straightforward and reliable solution to routinely perform preliminary data mining.

### 3.2. Method

The criteria^15^ defining the properties of the desired workflow are the following:

- Criteria for achieving genericity: applicability to small and big data, applicability to continuous or categorical or mixt data, and management of high dimensionality.
- Criteria for achieving ease of implementation and use: number of packages used, of algorithms used, of parameters to tune, use of “all-inclusive” packages covering at best the general clustering process.
- Criteria for achieving robustness and reliability: management of noise data, of multicollinearity, methods considered for clusters stability assessment, reliability of packages used (e.g., hosting site, renown, …), and reliability of algorithms used (e.g., renown, literature, …).

Facing the great diversity of packages^16^ and algorithms, and considering our goal of preserving the simplicity of implementation and use of the desired generic workflow, we focused on handy^17^ packages to cover the main algorithms and steps in the general clustering process (refer to Figure 1). Handy packages can include functions for clustering, cluster optimization, cluster evaluation, cluster stability evaluation, and cluster description (clustering algorithms suites). For Python, we considered the module *sklearn.cluster* from the scikit-learn library. For R, the following packages were selected: *FPC, cluster, clue*, and *clValid*. All these implementations, functions, and algorithms that compose them are considered robust and, therefore, **meet part of the criteria** for achieving robustness and reliability.

### 3.3. Preliminary work

When relevant, we matched the selected implementations to the defined criteria (refer to Table 1 for R packages and skikit-learn library, and Appendix A for clustering algorithms that compose them).

**Table 1:** Description of selected software implementations.

**Libraries**	**Language**	**Ease of implementation and use**				**Robustness**	**Number of downloads in 2021**
		**Data processing**	**Data clustering**	**Internal validation metrics**	**Clusters description**	**Cluster stability assessment**	
*FPC*	R	Yes	Yes	Silhouette width, Caliński-Harabasz index, Hubert's gamma coefficient, Dunn index, Tibshirani, and Walther's prediction strength, etc.	Yes	Bootstrap, noise, resampling, etc.	985,853
*Cluster*	R	Yes	Yes	Silhouette width, gap statistic, etc.	Yes	/	891,577
*Clue*	R	No	Yes	Variance accounted for (VEF), deviance accounted for (DEF), …	Yes	Bootstrap	467,260
*clValid*	R	No	Yes	Connectivity, Silhouette width, Dunn Index, etc.	Yes	Removing each column, one at a time	96,676
*sklearn.cluster*	Python	Yes	Yes	Rand index, Normalized Mutual Information (NMI), Adjusted Mutual Information (AMI), Silhouette width, Caliński-Harabasz Index, Davies-Bouldin Index, etc.	No	/	NA

Number of downloads in 2021 is based on the *cran_downloads()* function in the cranlogs R library. NA, not applicable.

Table 1 shows that neither the *cluster* R package nor the *sklearn-cluster* module in Python allows the evaluation of the stability of clusters. As indicated in Section 2, one should code this step oneself in Python, or link (if possible) with other packages in R. Of the selected R packages, *FPC* was the most downloaded in 2021 and provides the most internal assessment metrics. *Clue* and *FPC* evaluate cluster stability by bootstrapping but only *FPC* includes other methods such as noising, the complementarity of the two methods being recommended by Hennig (2008). *clValid* on the other hand proposes simpler methods, mainly used in biology, for evaluating the stability of clusters by removing the variables one by one.

The table in Appendix A is created based on Table 1 in Fahad et al. (2014), which we adapted for our purpose. Overall, Appendix A shows that none of the algorithms included in the selected packages satisfies all the properties sought in terms of genericity, simplicity of use and implementation, and robustness. For example, CLARA and Mini-batch *K*-means both allow very good handling of large data and are adapted to some extent to high dimensionality and rely on few parameters to be optimized. However, they only apply to continuous data and are not, particularly, suitable for noisy data. Also and unlike CLARA, Mini-batch *K*-means is only included in the scikit-learn module on Python.

This first synthesis work highlights the challenge to overcome.

### 3.4. Qluster

**Figure 3:**
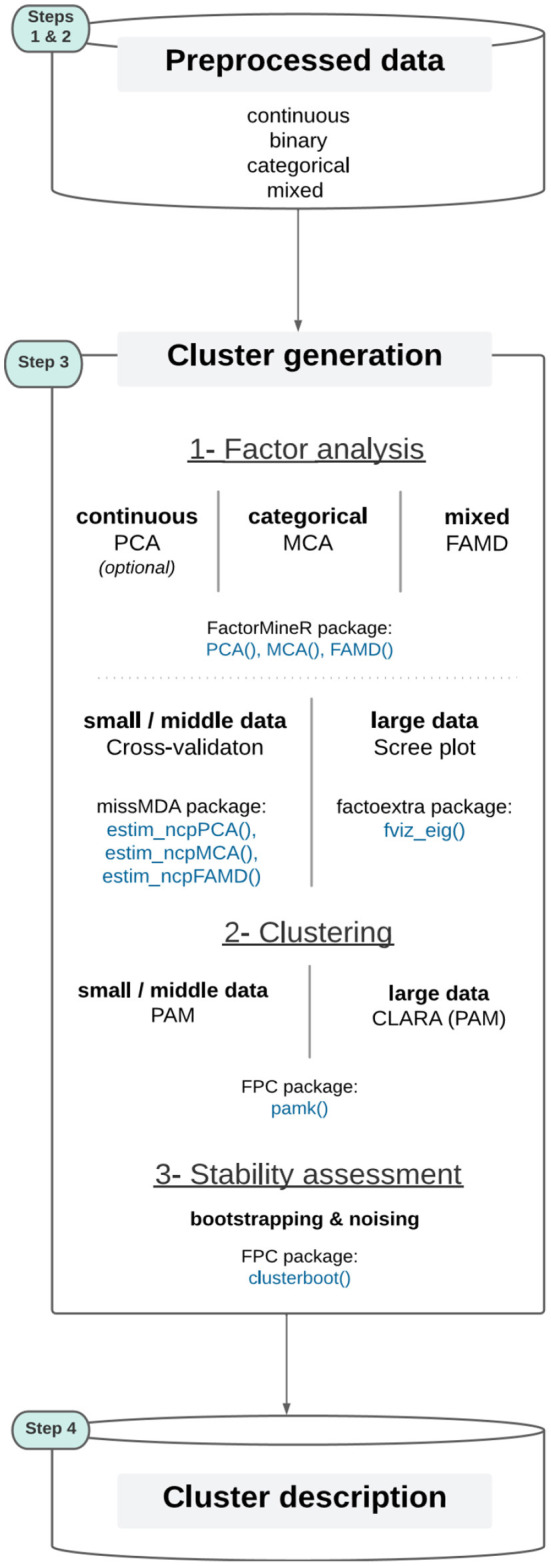
The Qluster workflow (the colored step pads correspond to those detailed in Figure 1).

Based on the literature review (Section 2) and the preliminary work (Section 3.3), we propose the *Qluster* workflow (refer to Figure 3), a set of methods that together represent a good balance for data scientists to make clustering on health data in a practical, efficient, robust and simple way. It covers the cluster generation step (step 3) through 1- factor analysis, 2- data clustering, and 3- stability evaluation. The output of the factor analysis (PCA, MCA, or FAMD) is the matrix of the coordinates of the individuals on the factorial dimensions, i.e., a table of continuous variables, allowing then the clustering by a PAM algorithm. For an in-depth discussion regarding the *Qluster* workflow, refer to Section 5.

To summarize, *Qluster* tries to generalize clustering tasks through a **generic** framework that is:

- **Adapted to variables of any nature**, be it categorical only, continuous only, or a mix of both. This is made possible by transforming all data in a continuous setting (that is both for mature in the literature and simpler to process) using methods for factor analysis [MCA for categorical data only or FAMD for mixed data (Pagès, 2004)]. As mentioned in Section 2.2, the latter also allows for dealing with **collinearity**, **high-dimensionality**, and **noise**. It also makes the work for a clustering algorithm easier, as there are both fewer variables to deal with and greater clarity in information to cluster (factor analysis methods are themselves meant to uncover profiles into components of richer information).
- **Adapted to datasets of any volume**, be it small or large data. Indeed, the same partitioning-based algorithm is used (PAM), either applied entirely on a dataset of reasonable size or on samples of a large^18^ dataset (CLARA algorithm), using the same *pamk()* function from the *FPC* R package. In addition to this **practical** aspect, PAM was chosen over the (widely used) *K*-means algorithm based on its ability to be deterministic and to deal with Manhattan distance, which is **less sensitive to outliers** than the Euclidean distance (Jin and Jiawei, 2010). Moreover, PAM is known for its **simplicity of use** [fewer parameters, e.g., than with DBSCAN or BIRCH (Fahad et al., 2014)] and is also implemented in an easy-to-use all-inclusive package (not the case for, e.g., CLARANS, KAMILA, Mini batch *K*-means, DENCLUE, and STING that are suited for large datasets but where assessing clusters stability would require extensive code development by data scientists). More details on the choice of the clustering algorithm can be found in section 5.

In addition, the *Qluster* workflow relies solely on four state-of-the-art R packages, allowing data scientists to quickly manage data of different natures and volumes and perform robust clustering:

- Both the clustering and cluster stability assessment tasks are performed using the *FPC* R package [functions *pamk()* and *clusterboot()*, respectively]. R has been chosen over Python because the former offers all the clustering methods desired, and no package including all the steps of interest to clustering was found for the latter (we would have to code some steps by ourself, refer to Section 2 for more details). The *clusterboot()* function offers many ways to assess clusters' stability, but one selects the two followings for routine practice and for their complementarity as mentioned in Hennig (2008): bootstrapping and noising.
- The factor analysis part is handled using the *FactoMineR* R package [functions *PCA(), MCA()*, and *FAMD()*, for continuous, categorical, and mixed data, respectively, the latter function generalizing the others]. This step is optional in the case where only continuous variables are in the input.^19^ To select the optimal number of components to keep, one recommends for small data to use the deterministic cross-validation technique implemented in the *missMDA* package [function *estim_ncpPCA(), estim_ncpMCA(), estim_ncpFAMD()* (Josse et al., 2012)]. As this method requires high computing time, the standard “elbow” method in a scree plot is recommended for large data, using the *factoextra* R package [function *fviz_eig()*].

Finally, the *Qluster* workflow is operationalizable and implementable from end to end (see in Appendix B a picture of implementation in the Dataiku^20^ platform. Available upon request: contact@quinten-france.com).

This generic workflow, usable in most situations, can be described through the following pseudocode (Algorithm 1):

**Algorithm 1:** The Qluster pseudo-code.

**input:** *X*: The input data
*packages*: FactoMineR, factoextra, FPC, missMDA
**output:** *Q*: A clustering of X and associated measures
1 **if** *X is continuous only* **then**
2 *F* = *PCA(X)*, with *F* a FactoMineR object of class PCA
3 **else if** *X is categorical only* **then**
4 *F* = *MCA(X)*, with *F* a FactoMineR object of class MCA
5 **else** // mixed continuous and categorical
6 *F* = *FAMD(X)*, with *F* a FactoMineR object of class FAMD
7 **end**
8 Define from *F* the matrix *M* of coordinates of individuals on each dimension
9 **if** *X is “large”* **then**
10 Apply *fviz_eig()* on *F* to select *C_*opt*_*, a sufficient number of components
11 Define the *M_*opt*_* matrix as *M* restricted to *C_*opt*_* components
12 *P* = *pamk(M_*opt*_)*, with *usepam* = FALSE (CLARA), *criterion* = “asw”, *scaling* = FALSE, and setting at convenience *samples, sampsize*, and *krange*
13 Let *K* the optimal number of clusters in *P*
14 **else** // X is not “large”
15 **if** *X is continuous only* **then**
16 *F_*ncp*_* = *estim_ncpPCA(X)*, with large [ncp.min, ncp.max] range
17 **else if** *X is categorical only* **then**
18 *F_*ncp*_* = *estim_ncpMCA(X)*, with large [ncp.min, ncp.max] range
19 **else** // mixed continuous and categorical
20 *F_*ncp*_* = *estim_ncpFAMD(X)*, with large [ncp.min, ncp.max] range
21 **end**
22 Retrieve the optimal number of dimensions *C_*opt*_* from *F_*ncp*_*
23 Define the *M_*opt*_* matrix as *M* restricted to *C_*opt*_* components
24 *P* = *pamk(M_*opt*_)*, with *usepam* = TRUE (PAM), *criterion* = “asw”, *scaling* = FALSE, and setting at convenience *krange*
25 Let *K* the optimal number of clusters in *P*
26 **end**
27 *S* = *clusterboot(M_*opt*_)*, with *bootmethod* = c(“boot”, “noise”), *krange* = *K, clustermethod* = “pamkCBI” and the same parameters as in the previous step. Loop on the *noise_level* parameter to test different noise levels
28 **Return** all useful results in *Q*

## 4. Detailed workflow methodology

### 4.1. The Cardiovascular disease dataset and the objective

The Cardiovascular Disease^21^ dataset includes 70,000 patients with or without cardiovascular disease and 12 variables (five of them are continuous).

The following raw variables were used (raw variables' names are in italic):

1. Age (days, converted into years) - *age*
2. Height (cm) - *height*
3. Weight (kg) - *weight*
4. Gender (M/F) - *gender*
5. Systolic blood pressure (SBP) (mmHg) - *ap_hi*
6. Diastolic blood pressure (DBP) (mmHg) - *ap_lo*
7. Cholesterol (categories 1: normal, 2: above normal, 3: well above normal) - *cholesterol*
8. Glucose (categories 1: normal, 2: above normal, 3: well above normal) - *gluc*
9. Smoking (Y/N) - *smoke*
10. Alcohol intake (Y/N) - *alco*
11. Physical activity (Y/N) - *active*
12. Presence or absence of cardiovascular disease (Y/N) - *cardio*

The objective of this section is to present in detail the application of the *Qluster* workflow proposed in Section 3 on the following use case: to characterize the phenotypes of patients with cardiovascular disease (a subset of patients with *cardio* = Y). This represents 34,979 patients (about 50% of the whole dataset).

### 4.2. Step-by-step application of the Qluster workflow

The following section details the application of the *Qluster* workflow to the cardiovascular dataset to help scientists use it for their own projects. Additional elements to the ones presented in Section 2 supporting the present methodology are also provided when relevant. We first present the preprocessing of the dataset, in which notably the few continuous variables are converted into qualitative data, before applying an MCA, which is a data-reduction technique for exploring the associations among multiple categorical variables (Greenacre, 1984; Warwick et al., 1989; Murtagh, 2005; Greenacre and Blasius, 2006; Nishisato, 2019). Then, given the large size of the database, the CLARA algorithm is applied and optimized. Finally, the clusters' stability is assessed and a brief interpretation of the clusters is provided.

#### 4.2.1. Data preparation

##### 4.2.1.1. Features derivation and selection

First, the Body Mass Index (BMI) variable was created from both height and weight (Ortega et al., 2016). Then, outliers were detected by defining for each quantitative variable the thresholds above or below which values are more likely to be inaccurate. Acceptable values should be in the following ranges: 18 ≤ Age <120, 10 ≤ BMI <100, SBP ≤ 400, and DBP ≤ 200 [Ortega et al., 2016; Mayo Clinic^22^, French HTA (HAS) recommendations^23^]. For simplicity, patients with at least one outlier were removed from the analysis (sensitivity analyses could be performed). Quantitative variables were then discretized in order to both create variables with a clinical sense and enable the use of the MCA algorithm (refer to Table 2).

**Table 2:** Description of quantitative feature engineering.

**Name**	**Description**	**Modalities**	**Accepted value range**
Age	Age	age ≤ 55; age > 55	[18, 120]
BMI	BMI	Underweight: <18.5 kg/m^2^; Normal: between 18.5 and 24.9 kg/m^2^; Overweight: between 25.0 and 29.9 kg/m^2^; Obese: ≥ 30.0 kg/m^2^	[10, 100]
*high_sbp*	High systolic blood pressure	1: SBP > 130 mmHg; 0: SBP ≤ 130 mmHg	≤ 400
*high_dbp*	High diastolic blood pressure	1: DBP > 80 mmHg; 0: DBP ≤ 80 mmHg	≤ 200

An additional binary *hypertension* variable was created based on both *high_sbp* and *high_dbp* variables that are used as a proxy for patients with hypertension [*hypertension* = 1 if *high_sbp* = 1 and *high_dbp* = 1; else *hypertension* = 0 (Williams et al., 2018)].

Finally, the variables selected to discriminate the patients must be chosen according to their medical relevance to the context of the study. To this end, the user must always consider the results he would obtain if a variable is included or not. In particular, the user has to ask himself whether active discrimination of the clusters by a variable is sought: considering the example of the two common variables age and race, if they are actively included in the clustering step, it will tend to create groups of young vs. old, Caucasian vs. non-Caucasian patients. If not, such variables can be kept for passive analysis of the generated clusters and assess *a posteriori* possible heterogeneity on these variables. In this use case, we removed the height, weight, and systolic and diastolic blood pressure features, as they are used to create the derived features listed above and are not useful alone for clustering.

At the end, we obtained a database of 34,134 patients and 11 variables.

##### 4.2.1.2. Dealing with low prevalent features and modalities

Clustering variables with low prevalence are known to be challenging in data analysis, especially for techniques that are very sensitive to data and/or anomalous cases [e.g., regression analysis and factor analysis (Fahrmeir et al., 2013)]. Most common techniques consist of either gathering rare modalities in groups of higher frequency or discarding the concerning modalities and/or variables. Additionally, binary clustering variables with low prevalence in the study population may be discarded from the analysis or grouped with other features when appropriate.

An arbitrary threshold of 10% was set to distinguish and eliminate features with rare modalities from the clustering features. This is consistent with recommendations before using Multiple Correspondence Analysis, which over-weighs rare modalities and multi-modality variables (Le Roux and Rouanet, 2010; Di Franco, 2016). When possible, modalities with <10% of prevalence were grouped with others based on medical relevance. As a result, both the Smoking (8.3% with *smoke* = Yes) and Alcohol intake (5.2% with *alco* = Yes) variables were ruled out to cluster data and were only used for *a posteriori* clusters description.

Moreover, some modalities were aggregated for the two following variables:

- Glucose (*gluc*): modalities 2 (above normal, 8.8%) and 3 (well-above normal, 9.5%) were grouped into one modality 2 (above normal).
- BMI (*BMI*): modalities “underweight” (0.5%) and “normal” were grouped into one modality “underweight & normal”.

At the end, the dataset used to perform MCA contains a total number of nine categorical variables (age, BMI, *high_sbp, high_dbp*, hypertension, gluc, gender, cholesterol, and physical activity).

#### 4.2.2. Perform multiple correspondence analysis

As with other methods for factor analysis (e.g., PCA and CA), MCA was combined with cluster analysis to capture data heterogeneity, through clusters of observations in the population that show distinctive patterns (Buuren and Heiser, 1989; Hwang et al., 2006; Mitsuhiro and Hiroshi, 2015; van de Velden et al., 2017; Testa et al., 2021).

The number of MCA components to be used was decided using the standard scree plot by identifying the “elbow” of the curve [method widely used with PCA (Cattell, 1996)] while constraining eigenvalues to be strictly above a threshold of 0.11 equivalent of Kaiser's rule in PCA (i.e., 1/*C* with *C* the number of categorical variables).

**Figure 4:**
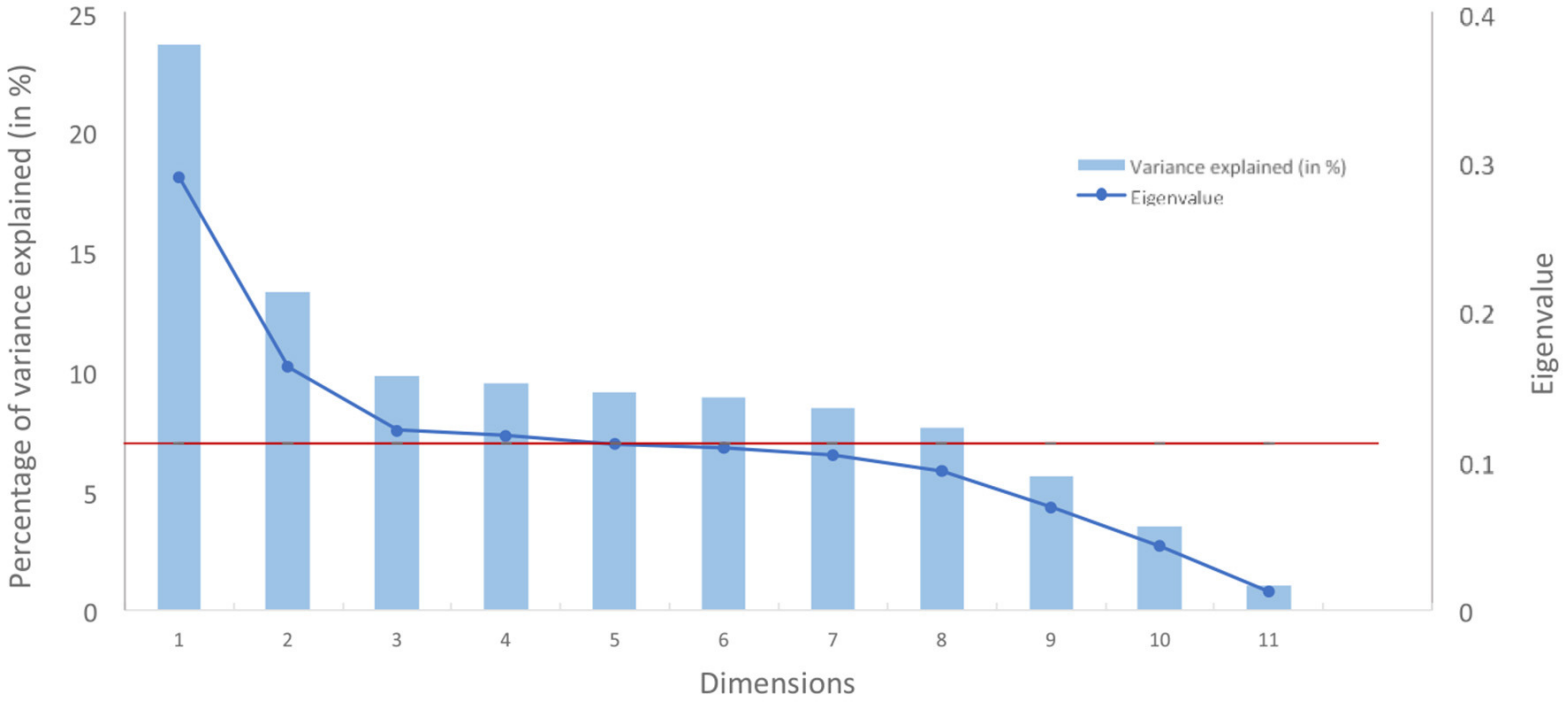
Scree plot of variance explained by dimension from an MCA (in red, the eigenvalue of 0.11).

Based on the scree plot (refer to Figure 4), three dimensions were chosen, the third marking a clear elbow in the curve (related eigenvalue: 0.12; related percentage of variance explained: 9.8%).

Moreover, and for interpretation purposes, eigenvalues were corrected using the Benzecri correction^24^ to consider that the binary coding scheme used in MCA creates artificial factors and, therefore, reduces the inertia explained (Greenacre, 1984). The top three components gather 99.9% of inertia after correcting with the Benzecri method (more details in Appendix D).

#### 4.2.3. Clustering of the data

##### 4.2.3.1. Parameters specification

The CLARA algorithm was used through the *pamk()* function in the *FPC* R package (version 2.2-5), a reliable package for flexible procedures for clustering, and with the following main parameters:

- Distance measure: dissimilarity matrix was computed using the Manhattan distance. The latter is more robust and less sensitive to outliers than the standard Euclidean distance (Jin and Jiawei, 2010).
- Number *K* of clusters: From 3–11,
  - The number of clusters was optimized on the Average Silhouette Width (ASW) quality measure, which is an internal validity metric reflecting the compactness and separation of the clusters. The ASW is based on the Silhouettes Width that was calculated for all patients in the best sample, i.e., the one used to obtain cluster medoids and generate clusters (Rousseeuw, 1987).
  - The range of clusters to be tested was determined to enable the identification of phenotypically similar subgroups while not generating an excessive number of subgroups for interpretation.

- The number of samples and sample size: 100 samples of 5% study population size (1,706 patients).
  - Experiments have shown that five samples of size 40 + 2C (with C the number of variables in input) give satisfactory results (Kaufman and Rousseeuw, 1990). However, increasing these numbers is recommended (if feasible), to limit sampling biases and favor converging toward the best solution. Equally, the higher the sample size is, the higher it is representative of the entire dataset. We, therefore, recommend pretesting on his material up to what parameter values the computation times are acceptable considering the size of the input dataset and the other steps of the workflow (including the clusters stability evaluation step, the most time consuming).

Other parameters include the non-scaling of the input data to not modify the observation space obtained from MCA.

##### 4.2.3.2. Results

**Figure 5:**
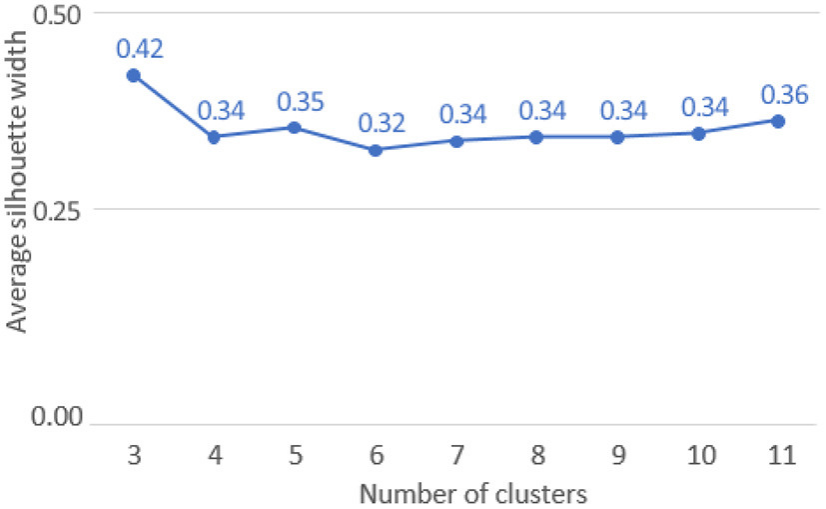
Average Silhouette Width for each set of clusters (*K* = 3–11).

The optimal ASW was obtained for a pool of three clusters (ASW: 0.42, refer to Figure 5).

Homogeneity and separability of clusters were further studied by analyzing the Silhouettes Width of patients in the best sample used to generate the clusters' medoids, using the *fviz silhouette()* function in the *factoextra* R package. As a reminder, the Silhouette Width characterizes both the cohesion of the cluster and its separation from the other clusters: a positive (respectively, negative) Silhouette Width for a patient is in favor of a correct (respectively, incorrect) affiliation to its own cluster.

**Figure 6:**
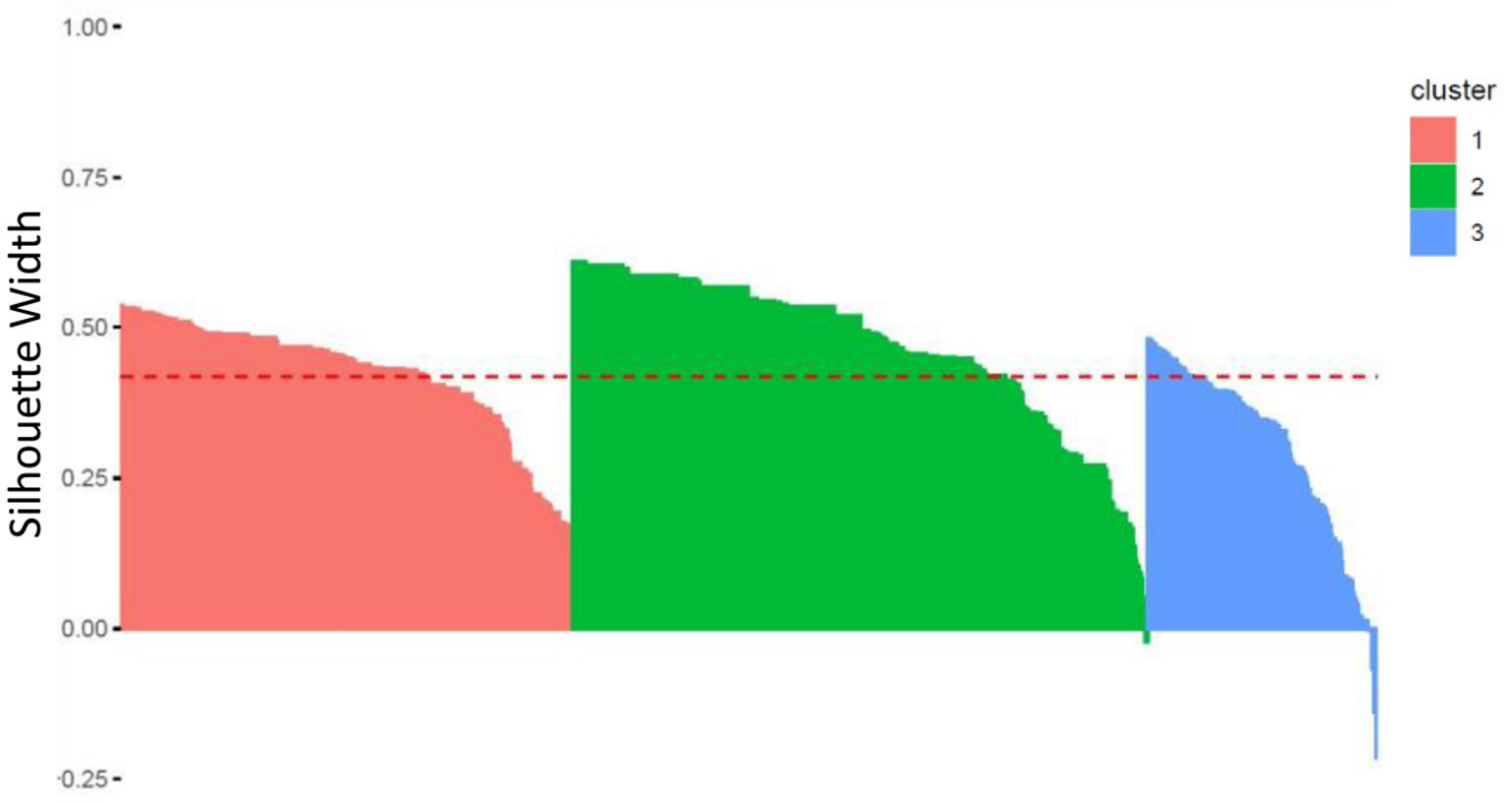
Silhouette Width for each patient within each of the three clusters (the red line corresponds to the Average Silhouette Width).

Figure 6 shows a high level of intra-cluster cohesion and inter-clusters separability as only few patients (in clusters 2 and 3) have negative Silhouettes. Clusterwise Silhouette Widths are also all positive (ASW of 0.42, 0.47, and 0.30, for clusters 1, 2, and 3, respectively).

#### 4.2.4. Cluster stability assessment

##### 4.2.4.1. Parameters specification

In order to evaluate clustering robustness, the clustering was performed several times on a cohort that was randomly modified. This allows generating under perturbations new versions of the original clusters and, thus, to evaluate the stability of the clusters to them. The stability of the clusters is all the higher as the new versions of the clusters generated under perturbations are similar to the original clusters. The data perturbation step was performed using two approaches that may provide complementary information based on the results in Hennig (2007): bootstrap and noise methods.

- Bootstrap approach:
  - This approach consists in performing the clustering as described in Section 4.2.3 on *B* = 50 bootstrapped data [i.e., random sampling with replacement (Efron, 1979; Efron and Tibshirani, 1994)], using the *clusterboot()* function in the *FPC* R package (version 2.2-5).
  - The Jaccard similarity metric is used to compute, for each cluster, the proximity between the clusters of patients obtained from the bootstrapped sample and the original clusters. It is given by the number of patients in common between the new cluster and the original cluster divided by the total number of distinct patients considered (i.e., present in either the new or the original cluster).
  - For each cluster, the following results are provided:
    ▪ the mean of the Jaccard similarity statistic.
    ▪ the number of times the cluster was “dissolved”, defined as a Jaccard similarity value ≤ 0.5. This value indicates instability.
    ▪ the number of times the cluster was “recovered”, defined as a Jaccard similarity value ≥ 0.75. This value indicates stability. There is some theoretical justification to consider a Jaccard similarity value smaller or equal to 0.5 as an indication of a “dissolved cluster”, refer to Hennig (2008). Between 0.6 and 0.75, clusters may be considered as indicating patterns in the data, but which points exactly should belong to these clusters is highly doubtful.

- Noise approach:
  - This approach consists in performing the clustering as described in Section 4.2.3 on *B* = 50 noisy data and for different values of noise, using the *clusterboot()* function in the *FPC* R package.
    ▪ Level of noise values: from 1 to 10%
  - The number of times each cluster was “dissolved” and “recovered” is provided, as well as the mean of the Jaccard similarity statistic, according to the noise values.

##### 4.2.4.2. Results

Clusters are all the more stable as the Jaccard similarity statistics and the number of recovered clusters are high, and the number of dissolved clusters is low.

The results of the data perturbation step are shown below:

- For the bootstrap approach, clusters 1, 2 and 3 all have a Jaccard similarity statistic of 100% over 50 iterations. The three clusters were recovered for 100% of bootstrapped iterations, which characterizes very high stability to resampling with replacement.
- For the noise approach, clusters 1, 2, and 3 at worst (for 2% noise) Jaccard similarity statistics of 100%, 98%, and 96%, respectively, over 50 iterations. The three clusters were recovered for 100% of iterations regardless of the level of noise (from 1 to 10%), which characterizes very high stability to noise.

Regardless of the method used, the results seem to be very robust and can certainly be explained by the large size of the database and the small number of clusters retained in the context of synthetic data. Clusters stability may be more variable in real cases.

It is worth noting that the *clusterboot()* function can also provide useful results and plots of clusters' stability (histogram of Jaccard similarity statistics by cluster, summary information for each cluster, etc.), but we did not provide them in this article since the obtained Jaccard similarity metrics were all around 100%.

#### 4.2.5. Clusters interpretation

Descriptive statistics (proportions and lift values) were computed from variables included or not in the clustering step. Cluster 1 [*n* = 12,272 (36.0%)] groups patients who all have high values of diastolic and systolic blood pressure, and consequently hypertension. These patients are slightly more than the average with well-above normal cholesterol values (18.5% vs. 17.7%) and above normal glucose values (20.0% vs. 18.3%). On the contrary, patients from clusters 2 and 3 [*n* = 15,477 (45.3%) and *n* = 6,385 (18.7%), respectively] are between 81% and 87% to have normal values of diastolic and systolic blood pressures, and none have hypertension. In contrast with cluster 2, patients from cluster 3 are many more with well-above normal cholesterol values (26.6% vs. 8.5%) and above normal glucose values (57.3% vs. 0.8%).

Patients from clusters 1 and 2 are overall younger than cluster 3 (age ≤ 55: 54.7% and 51.8% vs. 65.9%). Patients from clusters 1 and 3 are overall more obese than cluster 2 (41.4% and 44.5% vs. 21.8%).

To summarize, among patients with cardiovascular disease, cluster 1 gathers patients with hypertension, cluster 2 gathers patients healthier (although about the same age as cluster 1), and cluster 3 gathers slightly older patients with cholesterol and high levels of glucose (although no hypertension). Interestingly, the description of cluster 1 is consistent with a poorer lifestyle (lift values of 1.21 and 1.28 for Smoke and Alcohol, respectively) although this did not actively participate in clustering. Refer to Table 3 for more details.

**Table 3:** Prevalence and lift values of each modality and by cluster.

**Modalities**	**Prevalence (% of patients)**				**Lift values**		
	***C*1**	***C*2**	***C*3**	**Cohort**			
	***n* = 12,272**	***n* = 15,477**	***n* = 6,385**	***n* = 34,134**	**C1**	**C2**	**C3**
	**(36.0%)**	**(45.3%)**	**(18.7%)**	**(100%)**			
Female	62.1	64.2	71.4	64.8	0.96	0.99	1.10
Male	37.9	35.8	28.6	35.2	1.08	1.02	0.81
Cholesterol normal	60.6	90.7	16.5	66.0	0.92	1.37	0.25
Cholesterol above normal	20.8	8.5	26.6	16.3	1.28	0.52	1.63
Cholesterol well-above normal	18.5	0.7	56.9	17.7	1.05	0.04	3.22
Glucose normal	80.0	99.2	42.7	81.7	0.98	1.21	0.52
Glucose above normal	20.0	0.8	57.3	18.3	1.10	0.04	3.13
Physical activity	81.0	76.8	79.6	78.8	1.03	0.97	1.01
Age ≤ 55	45.3	48.2	34.1	44.5	1.02	1.08	0.77
Age > 55	54.7	51.8	65.9	55.5	0.99	0.93	1.19
BMI obese	41.4	21.8	44.5	33.1	1.25	0.66	1.34
BMI overweight	36.5	37.3	36.1	36.8	0.99	1.01	0.98
BMI normal or underweight	22.1	40.9	19.5	30.1	0.73	1.36	0.65
High Systolic blood pressure	100.0	14.3	18.5	45.9	2.18	0.31	0.40
High Diastolic blood pressure	100.0	12.9	16.8	44.9	2.23	0.29	0.37
Hypertension	100.0	0	0	36.0	2.78	0	0
Smoke	10.1	7.2	7.6	8.3	1.21	0.87	0.91
Alcohol	6.6	3.8	5.7	5.2	1.28	0.74	1.09

*C*1, *C*2, and *C*3 stand for Cluster 1, Cluster 2, and Cluster 3, respectively. Light blue: ≤ 0.5; blue: ≤ 1.0; yellow: ≤ 1.5; and green: >1.5. Lift is defined as % in the cluster vs. in the cohort.

## 5. Discussion

In this section, we will first discuss some limitations of the *Qluster* workflow and possible enhancements, then discuss choices of parameters and the practical use of this workflow.

### 5.1. Limitations and proposition for enhancing this workflow

#### 5.1.1. When large data are too large

As often in data mining, one limit concerns the size of the data. It is clear that for massive data, where the number of rows is very high, specific algorithms such as grid-based methods or canopy pre-clustering algorithms (McCallum et al., 2000) are needed for the algorithms to scale up.

More specifically, in such cases, factor analysis may be impossible to calculate, as it requires making matrix calculations and inverting matrix of size *n* ^*^ *p* (*n* individuals, *p* binary variables). Please note that in the case of categorical variables, one may prefer to use the anglo-saxon MCA method that applies the CA algorithm on a Burt table (*p* ^*^ *p*) instead of the complete disjunctive table (*n* ^*^ *p*), which is more efficient in computing time and, thus, more appropriate for large data [also implemented in the *MCA()* function in *FactoMineR* (Greenacre, 2007)]. Equally, in the case of very large data, the CLARA algorithm may be too time-consuming to compute as we still need to maintain enough samples and observations per sample for representativeness. For all these reasons, one suggests simply to analyze a random sample of the original dataset that is likely to be very representative of the latter while allowing the use of the *Qluster* workflow. Please also note that *PCA()* and *FAMD()* are known to take more time for computation than *MCA()*. One also suggests (when possible) to convert data into one type (continuous only or categorical only) in a data preparation step. Indeed, the upstream scaling of mixed data can be challenging, and the computation times by FAMD are more important. Alternatives may consist of not using the proposed workflow but algorithms that go fast on (very) large data such as Mini Batch *K*-means used on continuous variables or one-hot-encoded categorical variables. However, in addition to relying solely on Euclidean distance, these strategies may not allow for the prior use of factor analysis due to the size of the data, nor for the stability of clusters to be easily and properly assessed.

Conversely, when the number of columns is greater than the number of rows (*p* > *n*), the dimension reduction step *via* factor analysis methods makes even more sense to easily manage the high dimensionality of the data. However, in the most extreme cases where *p* >> *n*, standard factor methods may fail to yield consistent estimators of the loading vectors. In addition, the results may be difficult to interpret. In such situations, standardized methods may be a solution to improve the robustness, consistency, and interpretability of results (e.g., penalized PCA, Lee et al., 2012). It is also recommended that a relevant subset of the variables be selected prior to analysis (when possible).

#### 5.1.2. Generalizability of this workflow to missing data

Missing values management is not covered in this workflow, and it is, therefore, assumed that no missing values are present in the dataset. Indeed, both factor methods (PCA, MCA, FAMD) and proposed clustering methods (PAM, CLARA,…) require data without missing values. However, this workflow can be easily generalized to missing data, using the same *missMDA* package as for performing the selection of the optimal number of dimensions in factor analysis, in order to impute in a first step missing values using factor methods. The latter are state-of-the-art methods for handling missing values [e.g., function *imputePCA(), imputeMCA()*, and *imputeFAMD()* for simple imputations (Audigier et al., 2013)] and can, thus, easily be integrated and/or used in an automated workflow to handle missing data. In addition, this R package makes it possible to perform multiple imputations [*MIPCA(), MIMCA()*, and *MIFAMD()*] for assessing incertitudes from imputed values and increasing confidence in results (Josse and Husson, 2011). In this sense, the *Qluster* workflow can be easily modified to reach the state of the art in missing data management (refer to Appendix E for an example of the *Qluster* workflow adapted for handling missing values).

#### 5.1.3. Discussion on using factor analysis as a first step

Factor analysis allows the transformation of structured data of all kinds into continuous data, while dealing with large, collinear, noisy, and high-dimensional data. It also facilitates clustering by aggregating groups of homogeneous information within dimensions. Nevertheless, it cannot be guaranteed that the results will be “better” or “as good” with factor analysis in the clustering process. Similarly, the choice of factor analysis in this workflow comes with drawbacks that include the following items:

- The packages used cannot handle the ordinal nature of the variables. The latter must be treated as categorical or continuous.
- The observations × components matrix is continuous, although some raw variables could be categorical. This prevents the user from favoring (when relevant) positive co-occurrence over negative co-occurrence via the Jaccard similarity coefficient.

Alternatives may consist of data dimension reduction using feature selection methods, or manually, by grouping, transforming, and/or deleting variables based on clinical expertise.

#### 5.1.4. Discussion on using a single K-medoid algorithm

In order to provide a simple, yet generic and robust workflow for the practical use of the same methodology in many applications, we have made a careful selection of both algorithms and software packages. In particular, the decision to use the PAM/CLARA algorithm is based on many aspects such as the fact that it is:

- one of the best known, studied, and used algorithms by the community, for general purposes,
- suitable for continuous variables (i.e., the most mature in the literature).
- meant for the most frequent use case of clustering (i.e., hard partitioning),
- suitable for the Manhattan distance, a less sensitive to outliers distance, unlike its counterpart on the Euclidean distance (*K*-means),
- deterministic, due to its internal medoid initialization procedure, unlike the basic *K*-means algorithm that may lead to inconsistent or non-reproducible clusters,
- requiring few parameters to set up [e.g., conversely to BIRCH and DBSCAN, refer to Fahad et al. (2014)],
- very well-implemented within a recognized reference R package (the *FPC* package) facilitating its use within a complete and robust clustering approach,
- usable within the same R function [*pamk()*] regardless of the volume of data.

Yet, it is clear that other algorithms than the ones chosen could be routinely used, including those contained in the *FPC* R package to facilitate its integration within the workflow (e.g., DBSCAN and HAC). In particular, it is known that with non-flat geometry and/or uneven cluster size, DBSCAN is more appropriate than *K*-means and PAM. Equally, if the final goal is to obtain a hierarchy rather than a unique hard partition, the user may prefer an algorithm such as HAC, which can easily be used with the proposed packages in this workflow. However, the presence of additional parameters to tune or the lack of compatibility with massive data would make the workflow more complex. It is also important to note that this workflow is not intended to replace more in-depth work by data scientists to find what is optimal for a specific case study. More experienced data scientists can use the generic *Qluster* workflow for a first look at the data but are encouraged to adapt the general principles of this workflow to their case study (e.g., finding the most suitable algorithm). Such adaptations would be out-of-scope of this workflow in the sense of the initial objectives: genericity of applications while maintaining the simplicity of implementation and reliability/robustness of the methodology.

Equally, the user may want to benchmark several clustering algorithms as suggested by Hennig (2020). The comparison of methods solutions can be based on information measures (e.g., entropy and mutual information), internal validity measures (e.g., silhouette, refer to Section 2.4.), set-matching (i.e., mapping each cluster from the first clustering to the most similar cluster in the second clustering and computing recall, precision or any other measure), and pair counting [including dedicated visualization tools, refer to Achtert et al. (2012)]. Some of these strategies are directly implemented in the *clusterbenchstats()* function from the *FPC* R package or in the *clValid()* function of the *clValid* R package. However, as our goal is to propose a simple-to-use workflow, this complexification—which would also greatly impact computing times and memory capacities—is left to the user's discretion. Moreover, multiplying the algorithms and the combination of parameters forces one to rely more heavily on a purely statistical criterion (e.g., ASW) to select the “best” clustering of the data, although this may not reflect the best partitioning in a clinical sense. Indeed, ASW remains a criterion characterizing the average separability over all the clusters, and its optimum may miss (the set of) results that are clinically relevant and/or useful for the desired objective.^25^ If the data scientist wants to compare different algorithms, we recommend instead to fully explore the results of a well-chosen first algorithm, before challenging it with others, in order to be less dependent on the sole selection criterion of the ASW. This article, thus, takes the opposite view of the auto-ML literature by first advocating a full investigation of a parsimonious workflow made of well-chosen algorithms, rather than directly covering a wide range of algorithmic possibilities. On this topic, readers may be interested in recent areas of research around meta-clustering (Caruana et al., 2006) and ensemble clustering methods (Greene et al., 2004; Alqurashi and Wang, 2019). The first aims to produce several partitioning results so that the user can select those that are most useful. The second is intended to combine the clustering of several methods to propose a consensual result.

#### 5.1.5. Discussion on the clusters' stability assessment step

The bootstrapping and noise methods were chosen in the workflow because they are both available in the same function *clusterboot()* from the same package as for *pamk()*, and for their complementarity as recommended by Hennig (2007). Nevertheless, other methods may also be used as sensitivity analyses, including those proposed in the same *FPC* package. Furthermore, although this step allows for the clusters to be assessed, data scientists should keep in mind that stability is not the only important validity criterion—clusters obtained by very inflexible clustering methods may be stable but also not valid, as discussed in Hennig (2008). Finally, although several choices were made to try to manage outliers as best as possible, such as using a *K*-medoid algorithm and the Manhattan distance, the *Qluster* workflow does not fully address the issues related to outliers and extreme values. One solution may be to define threshold values to manually detect extreme values as a pre-processing step (as in the case study in Section 4), or to use more sophisticated statistical methods such as Yang et al. (2021).

#### 5.1.6. Discussion on the clusters' interpretation

Clusters' description is not covered in the *Qluster* workflow. However, many methods exist to interpret clusters (refer to Section 2.3). Data scientists can easily generalize *Qluster* to the description of clusters by using the functions already present in the *FPC* package in order not to make the workflow too complex, such as *plotcluster()* and *cluster.varstats()* following methodologies recommended by Hennig (2004).

#### 5.1.7. Discussion on the types of data that are supported by the Qluster workflow

Although general, the *Qluster* workflow does not cover all types of data and it is clear that for medical imaging data, omics data, or data in the form of signals, dedicated approaches must be considered. Nevertheless, most tabular data can be processed using the *Qluster* workflow. In this respect, although the *Qluster* workflow was specifically designed in the context of healthcare data analysis, it can easily be applied in other fields.

### 5.2. Discussion and recommendation on the practical use of the workflow

#### 5.2.1. Use of cluster stability as a criterion to be optimized

Cluster stability assessment could be considered as a criterion to be optimized, by iterating on this step in order to make this property an integral part of the clustering process itself. For example, stability measures could be used to select the optimal number of clusters, assuming that the clustering results are more stable with the correct number of clusters (Fränti and Rezaei, 2020).

However, attention should be paid to the fact that the bootstrap and noise methods are more computationally expensive than simple methods such as deleting the variables one by one (methods used on biological measurements and proposed in the *clValid* R package). Also, it may not be obvious to optimize the clustering on clusters' stability if the two proposed methods do not give similar results. For example, compared to the noise method, the bootstrap method is more likely to produce stable results as the size of the dataset increases in the case of PAM, and as the percentage of sample representativeness increases in the case of CLARA.

#### 5.2.2. What if the results are not satisfying?

The question of the ultimate relevance of clusters is not addressed in this workflow. It should be noted that the absence of results may be a result in itself, as it may characterize a population that cannot be described in terms of several homogeneous subgroups (either because such subgroups do not exist or because the variables used do not allow us to find them). Nevertheless, it is clear that, as in the Data Mining process, we can consider looping back on this workflow by changing certain parameters if the results are not satisfactory or if an important criterion of the clustering was not taken into account at the beginning (e.g., the maximum number of clusters). More generally, the data scientist is encouraged to keep in mind that the final objective of clustering is often the clinical relevance and usefulness of the results generated. In this sense and as mentioned in Section 5.1, it is not forbidden to relax a purely statistical criterion, such as the ASW (whose optimum may miss some relevant subgroups as it is an indicator of the overall separability) to better represent the diversity of the population studied, or to favor the generation of hypotheses in the case where the statistical optimum only gives broad results not enough specific for the initial objective.

In the same vein, negative silhouette values are viewed too pejoratively in the cluster validity analysis (interpreted as clustering failure). In fact, negative silhouettes characterize patients who, on average, are closer to patients from another cluster than to patients from their own cluster. Therefore, patients with a negative Silhouette may be informative of potential relationships between clusters and should, therefore, be considered as potential additional information about disease history and phenotypic complexity, such as one cluster that is the natural evolution of another. Hence, it is recommended that an analysis of patients with negative Silhouettes be included in the workflow to better assess whether they are a reflection of “bad” clustering or the key to better understanding the disease.

#### 5.2.3. What if the optimum number of clusters is the minimum of range *K*?

In the case where the optimal number of clusters is the minimum of the range of *K* (as in our example in Section 4), we recommend (if appropriate) that data scientists test for lower values of *K* to challenge the obtained optimum. Similarly, if the optimum is obtained for *K* = 2, data scientists should test whether the dataset should be split into two clusters, using the Duda–Hart test that tests the null hypothesis of homogeneity in the whole dataset. This can be done using the same *pamk()* function by setting up the minimum of *K* to 1, or directly using the *dudahart2()* function (also in the *FPC* R package). In any case, if the primary objective is to provide fine-grained knowledge of the study population, it will still be possible to provide results with the optimal *K* that was initially obtained, keeping in mind that the levels of inter-cluster separability and intra-cluster homogeneity are not really higher than those that would be obtained with a smaller number of clusters.

#### 5.2.4. Using this workflow routinely

The *Qluster* workflow can be easily automated for data scientists and organizations that need a routine way to cluster clinical data. Indeed, data scientists may create a main function for applying this workflow, including by setting the nature of data (categorical/continuous/mixed), the volume (normal/large), and parameters related to each called function. It is worth mentioning, however, that the quality of the input data and the structure of the groups to be found are factors that may not allow the present workflow to identify relevant results every time. In this case, the data scientist can refer to the indications given above or, if necessary, consider an approach more adapted to his data.

## 6. Conclusion

In this article, we propose *Qluster*, a practical workflow for data scientists because of its **genericity of application** (e.g., usable on small or big data, on continuous, categorical, or mixed variables, and database of high-dimensionality or not) while maintaining the **simplicity** of implementation and use (e.g., need for few packages and algorithms, few parameters to tune, …), and the **robustness and reliability** of the methodology (e.g., evaluation of the stability of clusters, use of proven algorithms and robust packages, and management of noisy or multicollinear data). It, therefore, does not rely on any innovative approach *per se* but rather on a careful selection and combination of state-of-the-art clustering methods for practical purposes and robustness.

Data clustering is a difficult task for many data scientists who are faced with a large literature and a large number of algorithms and implementations. We believe that *Qluster* can (1) **improve the quality of analyses** carried out as part of such studies (refer to *Qluster*'s criteria for robustness and reliability), **promote and ease clustering studies** (refer to *Qluster*'s criteria for genericity and simplicity of use), and **increase the skills** of some of the statisticians/data scientists involved (refer to the literature review provided and the general principles of *Qluster*). This workflow can also be used by more experienced data scientists for initial explorations of the data before designing more in-depth analyses.

Finally, this workflow can be fully operationalized, using either scripted tools or a Data Science platform supporting the use of R packages. As an illustrative example, we made an implementation on the Dataiku platform of the *Qluster* workflow to process a Kaggle dataset (refer to Appendix B). This implementation is usable on the free edition and is made available on request (email: contact@quinten-france.com).

## Data availability statement

Publicly available datasets were analyzed in this study. This data can be found here: https://www.kaggle.com/sulianova/cardiovascular-disease-dataset.

## Author contributions

MR and PG contributed very significantly throughout this study. CE and J-DZ were the main contributors to both the writing and the methodology. All authors participated in writing the manuscript, contributed to the revision of the manuscript, and read and approved the submitted version.

## Footnotes

^1^ https://www.kaggle.com/sulianova/cardiovascular-disease-dataset?select=cardio_train.csv

^2^For further details, see Celebi (2014).

^3^ https://scikit-learn.org/stable/

^4^ https://cran.r-project.org/web/packages/cluster/index.html

^5^ https://cran.r-project.org/web/packages/fpc/index.html

^6^ https://cran.r-project.org/web/packages/FactoMineR/index.html

^7^ https://github.com/MaxHalford/prince

^8^ https://cran.r-project.org/web/packages/missMDA/index.htmlpackages

^9^ https://cran.r-project.org/web/packages/factoextra/index.html

^10^ https://rdrr.io/github/QTCAT/qtcat/man/clarans.html

^11^ https://github.com/daveti/pycluster

^12^ https://pyclustering.github.io/

^13^ https://github.com/sandysa/Interpretable~Clustering

^14^ https://cran.r-project.org/web/packages/clValid/index.html

^15^A discussion on other possible criteria and ways to integrate them into the workflow is presented in Section 5.1 (e.g., management of missing data and outliers).

^16^e.g., CRAN Task View for cluster analysis: https://cran.r-project.org/web/views/Cluster.html.

^17^e.g. of handy R packages: https://towardsdatascience.com/a-comprehensive-list-of-handy-r-packages-e85dad294b3d.

^18^The notion of large data, as well as how to fix the hyperparameters *samples* and *sampsize* in CLARA algorithm, may vary according to the computing capabilities of the user's system. One recommends users to pre-tests different scenarios to adapt these thresholds to their own settings. For guidance, this workflow applied on the case study in Section 4 (34,134 observations and nine variables) took 5 h and 30 min with 8 CPUs and 10 GB RAM.

^19^It is worth noting that standardization of continuous data is recommended before using PCA, to not give excessive importance to the variables with the largest variances.

^20^ https://www.dataiku.com/

^21^On Kaggle: www.kaggle.com/sulianova/cardiovascular-disease-dataset

^22^ https://www.mayoclinic.org/diseases-conditions/high-blood-pressure/diagnosis-treatment/drc-20373417#:%CB%9C:text=Your%20blood%20pressure%20below%2080%20mm%20Hg

^23^Prise en charge des patients adultes atteints d'hypertension arterielle essentielle - Actualisation 2005. https://www.has-sante.fr/upload/docs/application/pdf/2011-09/hta_2005-recommandations.pdf.

^24^One may prefer the Greenacre adjustment to Benzecri correction, which tends to be less optimistic than the Benzecri correction. Please note that both methods are not currently implemented in the proposed R packages and must therefore be implemented by data scientists if desired. R code for Benzecri correction is provided in Appendix C.

^25^Similarly, the user may want to compare several methods for selecting the optimal number of clusters, including other direct methods (e.g., elbow method on the total within-cluster sum of square) or methods based on statistical testing (e.g., gap statistic).

## Supplementary material

Appendix A Description of algorithms in selected libraries on some of the criteria defining genericity, ease of implementation and use, and robustness.

Appendix B Example of an implementation of the Qluster workflow on the Dataiku platform.

Appendix C R Code to implement Benzecri correction from MCA eigenvalues.

Appendix D Eigenvalues and variances explained with and without Benzecri correction.

Appendix E Example of the Qluster workflow adapted for handling missing values.

## References

[B1] Achtert E. Goldhofer S. Kriegel H.-P. Schubert E. Zimek A. (2012). “Evaluation of clusterings – metrics and visual support,” *2012 IEEE 28th International Conference on Data Engineering* (Arlington, VA), 1285–1288. 10.1109/ICDE.2012.128

[B2] Ahmad A. Khan S. S. (2019). Survey of state-of-the-art mixed data clustering algorithms. IEEE Access 7, 31883–31902. 10.1109/ACCESS.2019.2903568

[B3] Aljalbout E. Golkov V. Siddiqui Y. Strobel M. Cremers D. (2018). Clustering 0with deep learning: taxonomy and new methods. arXiv:1801.07648. 10.48550/arXiv.1801.07648

[B4] Alqurashi T. Wang W. (2019). Clustering ensemble method. Int. J. Mach. Learn. Cyber. 10, 1227–1246. 10.1007/s13042-017-0756-7

[B5] Altman N. Krzywinski K. (2017). Clustering. Nat. Methods 14, 545–546. 10.1038/nmeth.4299

[B6] Arabie P. Hubert L. (1994). Cluster analysis in marketing research. Adv. Methods Market. Res. 160−189.

[B7] Arbelaitz O. Gurrutxaga I. Muguerza J. Pérez J. M. Perona I. (2013). An extensive comparative study of cluster validity indices. Pattern Recognit. 46, 243–256. 10.1016/j.patcog.2012.07.02117217348

[B8] Arthur D. Vassilvitskii S. (2007). “k-means++: the advantages of careful seeding,” in Proceedings of the Eighteenth Annual ACM-SIAM Symposium on Discrete Algorithms (Philadelphia, PA: Society for Industrial and Applied Mathematics), 1027–1035.

[B9] Audigier V. Husson H. Josse J. (2013). A principal components method to impute missing values for mixed data. arXiv:1301.4797. 10.1007/s11634-014-0195-1

[B10] Bandalos D. L. Boehm-Kaufman M. R. (2010). “Four common misconceptions in exploratory factor analysis,” in Statistical and Methodological Myths and Urban Legends (Routledge), 81–108.

[B11] Bertsimas D. Orfanoudaki A. Wiberg H. (2021). Interpretable clustering: an optimization approach. Mach. Learn. 110, 89–138. 10.1007/s10994-020-05896-2

[B12] Bezdek J. C. Pal N. R. (1998). Some new indexes of cluster validity. IEEE Transact. Syst. Man Cybernet. 28, 301–315. 10.1109/3477.678624 18255949

[B13] Bezdek J. C. Robert E. William F. (1984). FCM: the fuzzy c-means clustering algorithm. Comput. Geosci. 10, 191–203. 10.1016/0098-3004(84)90020-735836918

[B14] Bock H. H. (1987). “On the Interface between Cluster Analysis, Principal Component Analysis, and Multidimensional Scaling,” in Multivariate Statistical Modeling and Data Analysis: Proceedings of the Advanced Symposium on Multivariate Modeling and Data Analysis May 15–16, 1986, eds H. Bozdogan, and A. K. Gupta (Dordrecht: Springer Netherlands; Theory and Decision Library), 17–34.

[B15] Bousquet P. J. Philippe D. Abir T. Kamal M. Pascal D. Jean B. (2015). Clinical relevance of cluster analysis in phenotyping allergic rhinitis in a real-life study. Int. Arch. Allergy Immunol. 166, 231–240. 10.1159/000381339 25924687

[B16] Bro R. Kjeldahl K. Smilde A. K. Kiers H. A. L. (2008). Cross-validation of component models: a critical look at current methods. Anal. Bioanal. Chem. 390, 1241–1251. 10.1007/s00216-007-1790-1 18214448

[B17] Brock G. Vasyl P. Susmita D. Somnath D. (2008). clValid: An R Package for Cluster Validation. Available online at: https://www.jstatsoft.org/article/view/v025i04 (accessed December 16, 2021).

[B18] Buuren S. Heiser W. J. (1989). Clusteringn objects intok groups under optimal scaling of variables. Psychometrika 54, 699–706. 10.1007/BF02296404

[B19] Caliński T. Harabasz J. (1974). A dendrite method for cluster analysis. Commun. Stat. 3, 1–27. 10.1080/03610927408827101

[B20] Caruana R. Elhawary M. Nguyen N. Smith C. (2006). “Meta clustering,” in Sixth International Conference on Data Mining (ICDM'06) (Hong Kong), 107–118. 10.1109/ICDM.2006.103

[B21] Cattell R. B. (1996). The scree test for the number of factors. Multivariate Behav. Res. 1, 245–276. 10.1207/s15327906mbr0102_10 26828106

[B22] Celebi M. E. (ed.). (2014). Partitional Clustering Algorithms. Springer. 10.1007/978-3-319-09259-1

[B23] Ciampi A. Yves L. (2000). “Clustering large, multi-level data sets: an approach based on kohonen self organizing maps,” in Principles of Data Mining and Knowledge Discovery, Lecture Notes in Computer Science, eds D. A. Zighed, J. Komorowski, and J. Zytkow (Berlin, Heidelberg: Springer), 353–358.

[B24] Clausen S. E. (1998). Applied Correspondence Analysis: An Introduction. Sage. Available online at: https://en.wikipedia.org/w/index.php?title=Cluster_analysis&oldid=1034255231 (accessed July 18, 2021).

[B25] Costa P. S. Santos N. C. Cunha P. Cotter J. Sousa N. (2013). The Use of Multiple Correspondence Analysis to Explore Associations between Categories of Qualitative Variables in Healthy Ageing. Available online at: https://www.hindawi.com/journals/jar/2013/302163/ (accessed December 16, 2021). 10.1155/2013/302163 PMC3810057 24222852

[B26] Datta S. Somnath D. (2006). Methods for evaluating clustering algorithms for gene expression data using a reference set of functional classes. BMC Bioinformat. 7, 397. 10.1186/1471-2105-7-397 16945146 PMC1590054

[B27] De Soete G. Carroll J. D. (1994). “K-means clustering in a low-dimensional Euclidean space,” in New Approaches in Classification and Data Analysis, eds E. Diday, Y. Lechevallier, M. Schader, P. Bertrand, and B. Burtschy (Berlin, Heidelberg: Springer; Studies in Classification, Data Analysis, and Knowledge Organization), 212–219.

[B28] DeSarbo W. S. Daniel J. H. Kamel J. (1991). Multiclus: a new method for simultaneously performing multidimensional scaling and cluster analysis. Psychometrika 56, 121–136. 10.1007/BF02294590

[B29] Di Franco G. (2016). Multiple correspondence analysis: one only or several techniques? Qual. Quant. 50, 1299–1315. 10.1007/s11135-015-0206-0

[B30] Do C. B. Serafim B. (2008). What is the expectation maximization algorithm? Nat. Biotechnol. 26, 897–899. 10.1038/nbt1406 18688245

[B31] Drennan R. D. (2010). Statistics for Archaeologists. New York, NY: Springer. 10.1007/978-1-4419-0413-3

[B32] Efron B. (1979). Bootstrap methods: another look at the jackknife. Ann. Stat. 7, 1–26. 10.1214/aos/1176344552

[B33] Efron B. Tibshirani R. J. (1994). An Introduction to the Bootstrap. CRC Press, 456.

[B34] Esnault C. May-Line G. Maxence Q. Alexandre T. Jean-daniel Z. (2020). Q-Finder: an algorithm for credible subgroup discovery in clinical data analysis - an application to the international diabetes management practice study. Front. Artif. Intell. 3, 559927. 10.3389/frai.2020.559927 33733209 PMC7861304

[B35] Ester M. Hans-Peter K. Jorg S. Xiaowei X. (1996). “A density-based algorithm for discovering clusters in large spatial databases with noise,” in Proceedings of the Second International Conference on Knowledge Discovery and Data Mining. KDD'96 (Portland, OR: AAAI Press), 226–231.

[B36] Estivill-Castro V. (2002). Why so many clustering algorithms: a position paper. ACM SIGKDD Explorat. Newslett. 4, 65–75. 10.1145/568574.568575

[B37] Fahad A. Najlaa A. Zahir T. Abdullah A. Ibrahim K. Albert Y. Z.. (2014). A survey of clustering algorithms for big data: taxonomy and empirical analysis. IEEE Transact. Emerg. Topi. Comput. 2, 267–279. 10.1109/TETC.2014.2330519

[B38] Fahrmeir L. Thomas K. Stefan L. Brian M. (2013). “Categorical regression models,” in Regression: Models, Methods and Applications, eds L. Fahrmeir, T. Kneib, S. Lang, and B. Marx (Berlin, Heidelberg: Springer), 325–347.

[B39] Fisher D. H. (1987). Knowledge acquisition via incremental conceptual clustering. Mach. Learn. 2, 139–172. 10.1007/BF00114265

[B40] Foss A. H. Marianthi M. (2018). Kamila: clustering mixed-type data in R and hadoop. J. Stat. Softw. 83, 1–44. 10.18637/jss.v083.i13

[B41] Fränti P. Rezaei M. (2020). Can the Number of Clusters Be Determined by External Indices? Available online at: https://ieeexplore.ieee.org/document/9090190 (accessed May 12, 2022).

[B42] Fränti P. Sieranoja S. Wikström K. Laatikainen T. (2022). Clustering diagnoses from 58 million patient visits in finland between 2015 and 2018. JMIR Med. Informat. 10, e35422. 10.2196/35422 35507390 PMC9118010

[B43] Gordon A. D. (1999). Classification - 2nd Edn. Routledge Book. Available online at: https://www.routledge.com/Classification/Gordon/p/book/9780367399665 (accessed December 15, 2021).

[B44] Green P. E. Abba M. K. (1995). A comparison of alternative approaches to cluster-based market segmentation. Mark. Res. Soc. J. 37, 1–19. 10.1177/147078539503700302

[B45] Greenacre M. (2007). Correspondence Analysis in Practice. 2nd Edn. New York, NY: Chapman and Hall/CRC.

[B46] Greenacre M. Blasius J. (eds.). (2006). Multiple Correspondence Analysis and Related Methods. New York, NY: Chapman and Hall/CRC.

[B47] Greenacre M. J. (1984). Theory and Applications of Correspondence Analysis. Academic Press.

[B48] Greene D. Tsymbal A. Bolshakova N. Cunningham P. (2004). “Ensemble clustering in medical diagnostics,” in Proceedings. 17th IEEE Symposium on Computer-Based Medical Systems (Bethesda, MD), 576–581. 10.1109/CBMS.2004.1311777

[B49] Guha S. Rajeev R. Kyuseok S. (1998). CURE: an efficient clustering algorithm for large databases. ACM SIGMOD Rec. 27, 73–84. 10.1145/276305.276312

[B50] Guha S. Rajeev R. Kyuseok S. (2000). Rock: A Robust Clustering Algorithm for Categorical Attributes. Available online at: https://www.sciencedirect.com/science/article/abs/pii/S0306437900000223 (accessed December 15, 2000).

[B51] Halkidi M. Yannis B. Michalis V. (2001). On clustering validation techniques. J. Intell. Inf. Syst. 17, 107–145. 10.1023/A:1012801612483

[B52] Handl J. Joshua K. Douglas B. K. (2005). Computational cluster validation in postgenomic data analysis. Bioinformatics 21, 3201–3212. 10.1093/bioinformatics/bti517 15914541

[B53] Hennig C. (2004). Asymmetric linear dimension reduction for classification. J. Comput. Graph. Statist. 13, 930–945. 10.1198/106186004X12740

[B54] Hennig C. (2007). Cluster-Wise Assessment of Cluster Stability. Available online at: https://www.sciencedirect.com/science/article/abs/pii/S0167947306004622?via%3Dihub (accessed November 9, 2021).

[B55] Hennig C. (2008). Dissolution point and isolation robustness: robustness criteria for general cluster analysis methods. J. Multivariat. Anal. 99, 1154–1176. 10.1016/j.jmva.2007.07.002

[B56] Hennig C. (2014). “How many bee species? A case study in determining the number of clusters,” in Data Analysis, Machine Learning and Knowledge Discovery, eds M. Spiliopoulou, L. SchmidtThieme, and R. Janning (Cham: Springer International Publishing; Studies in Classification, Data Analysis, and Knowledge Organization), 41–49.

[B57] Hennig C. (2020). Cluster validation by measurement of clustering characteristics relevant to the user. arXiv:1703.09282. 10.1002/9781119597568.ch1

[B58] Hennig C. Liao T. F. (2013). How to find an appropriate clustering for mixed-type variables with application to socio-economic stratification. J. R. Stat. Soc. Ser. C. 62, 309–369. 10.1111/j.1467-9876.2012.01066.x

[B59] Hinneburg A. Keim D. A. (1998). An Efficient Approach to Clustering in Large Multimedia Databases With Noise, Vol. 98. Bibliothek der Universität Konstanz, 58–65. Available online at: https://www.aaai.org/Papers/KDD/1998/KDD98-009.pdf

[B60] Huang Z. (1997). A fast clustering algorithm to cluster very large categorical data sets in data mining. DMKD. 3, 34–39.

[B61] Huang Z. (1998). Extensions to the k-means algorithm for clustering large data sets with categorical values. Data Min. Knowl. Discover. 2, 283–304. 10.1023/A:1009769707641

[B62] Hwang H. Dillon W. R. Takane Y. (2006). An extension of multiple correspondence analysis for identifying heterogeneous subgroups of respondents. Psychometrika 71, 161–171. 10.1007/s11336-004-1173-x

[B63] Jain A. (2010). Data clustering: 50 years beyond K-means. Pattern Recogn. Lett. 31, 651–666. 10.1016/j.patrec.2009.09.01131125497

[B64] Jin X. Jiawei H. (2010). “K-medoids clustering,” in Encyclopedia of Machine Learning, eds C. Sammut, and G. I. Webb (Boston, MA: Springer), 564–565.

[B65] Josse J. Chavent M. Liquet B. Husson F. (2012). Handling missing values with regularized iterative multiple correspondence analysis. J. Classif. 29, 91–116. 10.1007/s00357-012-9097-0

[B66] Josse J. Husson F. (2011). Multiple imputation in principal component analysis. Adv. Data Anal. Classif. 5, 231–246. 10.1007/s11634-011-0086-7

[B67] Kamoshida R. Fuyuki I. (2020). “Automated clustering and knowledge acquisition support for beginners,” in Procedia Computer Science. Knowledge-Based and Intelligent Information & Engineering Systems: Proceedings of the 24th International Conference KES2020, 1596–1605. Available online at: https://www.sciencedirect.com/science/article/pii/S1877050920320846 (accessed September 9, 2021).

[B68] Kaufman L. Rousseeuw P. J. (1990). “Partitioning around medoids (Program PAM),” in Finding Groups in Data. Section: 2 eprint (John Wiley & Sons, Ltd.), 68–125. Available online at: https://onlinelibrary.wiley.com/doi/pdf/10.1002/9780470316801.ch2; https://onlinelibrary.wiley.com/doi/abs/10.1002/9780470316801.ch2 (accessed December 5, 2021).

[B69] Kaufman L. Rousseeuw P. J. (2009). Finding Groups in Data: An Introduction to Cluster Analysis. John Wiley & Sons.

[B70] Kaushik M. Bhawana M. (2014). Comparative study of K-means and hierarchical clustering techniques. Int. J. Softw. Hardw. Res. Eng. 2, 93–98.

[B71] Kiselev V. Y. Tallulah S. A. Martin H. (2019). Challenges in unsupervised clustering of single-cell RNA-seq data. Nat. Rev. Genet. 20, 273–282. 10.1038/s41576-018-0088-9 30617341

[B72] Kleinberg J. (2002). “An impossibility theorem for clustering,” in Proceedings of the 15th International Conference on Neural Information Processing Systems (NIPS'02) (Cambridge, MA: MIT Press), 463–470.

[B73] Lange T. Volker R. Mikio L. B. Joachim B. (2004). Stability-based validation of clustering solutions. Neural Comput. 16, 1299–1323. 10.1162/089976604773717621 15130251

[B74] Le Roux B. Rouanet H. (2010). Multiple Correspondence Analysis, Vol. 163. Sage. 10.4135/9781412993906

[B75] Lee Y. K. Lee E. R. Park B. U. (2012). Principal component analysis in very high-dimensional spaces. Stat. Sin. 22, 933–956. 10.5705/ss.2010.14915971925

[B76] Li N. Latecki L. J. (2017). “Affinity learning for mixed data clustering,” in IJCAI, 2173–2179. Available online at: https://www.ijcai.org/Proceedings/2017/0302.pdf

[B77] Lorenzo-Seva U. (2011). Horn's parallel analysis for selecting the number of dimensions in correspondence analysis. Methodology 7, 96–102. 10.1027/1614-2241/a000027

[B78] MacQueen J. (1967). “Some methods for classification and analysis of multivariate observations,” in Proceedings of the Fifth Berkeley Symposium on Mathematical Statistics and Probability, Volume 1: Statistics (University of California Press), 281–298. Available online at: https://projecteuclid.org/ebooks/berkeley-symposium-on-mathematical-statistics-and-probability/Proceedings-of-the-Fifth-Berkeley-Symposium-on-Mathematical-Statistics-and/chapter/Some-methods-for-classification-and-analysis-of-multivariate-observations/bsmsp/1200512992 (accessed December 5, 2021).

[B79] McCallum A. Kamal N. Lyle H. U. (2000). “Efficient clustering of high-dimensional data sets with application to reference matching,” in KDD '00: Proceedings of the Sixth ACM SIGKDD International conFerence on Knowledge Discovery and Data Mining (Boston, MA: ACM Press), 169–178.

[B80] McCane B. Michael A. (2008). Distance functions for categorical and mixed variables. Pattern Recognit. Lett. 29, 986–993. 10.1016/j.patrec.2008.01.02124368765

[B81] McInnes L. Healy J. Melville J. (2018). Umap: uniform manifold approximation and projection for dimension reduction. arXiv 1802, 03426. 10.21105/joss.00861

[B82] Meilă M. (2007). Comparing clusterings—an information based distance. J. Multivar. Anal. 98, 873–895. 10.1016/j.jmva.2006.11.013

[B83] Milligan G. W. Martha C. C. (1985). An examination of procedures for determining the number of clusters in a data set. Psychometrika 50, 159–179. 10.1007/BF0229424517825091

[B84] Mitsuhiro M. Hiroshi Y. (2015). Reduced k-means clustering with MCA in a lowdimensional space. Comput. Stat. 30, 463–475. 10.1007/s00180-014-0544-8

[B85] Mittal M. Lalit M. G. Duraisamy J. H. Jasleen K. S. (2019). Clustering approaches for high-dimensional databases: a review. Wiley Interdiscipl. Revi. Data Mining Knowl. Discov. 9, e1300. 10.1002/widm.1300

[B86] Murtagh F. (2005). Correspondence Analysis and Data Coding With Java and R. Chapman and Hall/CRC. 10.1201/9781420034943

[B87] Nagpal A. Jatain A. Gaur D. (2013). “Review based on data clustering algorithms,” in 2013 IEEE Conference on Information & Communication Technologies (IEEE), 298–303. 10.1109/CICT.2013.6558109

[B88] Ng R. Jiawei H. (2002). CLARANS: a method for clustering objects for spatial data mining. IEEE Trans. Knowl. Data Eng. 14, 1003–1016. 10.1109/TKDE.2002.1033770

[B89] Ng R. T. Han J. (1994). “Efficient and effective clustering methods for spatial data mining,” in Proceedings of VLDB, 144–155.31029259

[B90] Nietto P. R. Maria C. N. (2017). “Estimating the number of clusters as a preprocessing step to unsupervised learning,” in Intelligent Systems Design and Applications. Advances in Intelligent Systems and Computing, eds A. M. Madureira, A. Abraham, D. Gamboa, and P. Novais (Cham: Springer International Publishing), 25–34.

[B91] Nishisato S. (2019). Analysis of Categorical Data: Dual Scaling and its Applications. University of Toronto Press. Available online at: https://www.degruyter.com/document/doi/10.3138/9781487577995/html (accessed December 5, 2021).

[B92] Obembe O. Oyelade J. (2019). Data Clustering: Algorithms and Its Applications. IEEE Xplore. Available online at: https://ieeexplore.ieee.org/document/8853526 (accessed March 16, 2021).

[B93] Ortega F. B. Carl J. L. Steven N. B. (2016). Obesity and cardiovascular disease. Circ. Res. 118, 1752–1770. 10.1161/CIRCRESAHA.115.306883 27230640

[B94] Oyelade J. Isewon I. Oladipupo O. Emebo O. Omogbadegun Z. Aromolaran O.. (2019). “Data clustering: Algorithms and its applications” in 2019 19th International Conference on Computational Science and Its Applications (ICCSA) (St. Petersburg), 71–81. 10.1109/ICCSA.2019.000-1

[B95] Pagès J. (2004). Analyse Factorielle de Donnees Mixtes. Available online at: http://www.numdam.org/article/RSA_2004__52_4_93_0.pdf (accessed March 19, 2021).

[B96] Pagès J. Husson F. (2017). Exploratory Multivariate Analysis by Example Using R 2nd Edition - F. Available online at: https://www.routledge.com/Exploratory-Multivariate-Analysis-by-Example-Using-R/Husson-Le-Pages/p/book/9780367658021 (accessed December 16, 2021).

[B97] Rezaei M. Pasi F. (2016). Set matching measures for external cluster validity. IEEE Trans. Knowl. Data Eng. 28, 2173–2186. 10.1109/TKDE.2016.2551240

[B98] Rousseeuw P. J. (1987). Silhouettes: a graphical aid to the interpretation and validation of cluster analysis. J. Comput. Appl. Math. 20, 53–65. 10.1016/0377-0427(87)90125-7

[B99] Saint Pierre A. Giemza J. Alves I. Karakachoff M. Gaudin M. Amouyel P. (2020). The genetic history of France. Eur. J. Hum. Genet. 28, 853–865. 10.1038/s41431-020-0584-1 32042083 PMC7316781

[B100] Saisubramanian S. Sainyam G. Shlomo Z. (2020). Balancing the tradeoff between clustering value and interpretability. arXiv:1912.07820. 10.1145/3375627.3375843

[B101] Sculley D. (2010). “Web-scale k-means clustering,” in Proceedings of the 19th International Conference on World Wide Web. WWW '10 (New York, NY: Association for Computing Machinery), 1177–1178.

[B102] Sheikholeslami G. Chatterjee S. Zhang A. (1998). WaveCluster: A MultiResolution Clustering Approach for Very Large Spatial Databases — Semantic Scholar. Available online at: https://www.semanticscholar.org/paper/WaveCluster%5C%3A-A-Multi-Resolution-Clustering-Approach-Sheikholeslami-Chatterjee/f0015f0e834a84699a9b83c6c9af33acdac05069 (accessed December 16, 2021).

[B103] Shirkhorshidi A. S. Aghabozorgi S. Wah T. Y. Herawan T. (2014). “Big data clustering: A review,” in Computational Science and Its Applications - ICCSA 2014. ICCSA 2014. Lecture Notes in Computer Science, Vol. 8583. Cham: Springer. 10.1007/978-3-319-09156-3_4933816971

[B104] Sieranoja S. Pasi F. (2019). Fast and general density peaks clustering. Pattern Recognit. Lett. 128, 551–558. 10.1016/j.patrec.2019.10.01920932336

[B105] Sieranoja S. Pasi F. (2022). Adapting k-means for graph clustering. Knowl. Inf. Syst. 64, 115–142. 10.1007/s10115-021-01623-y

[B106] Takane Y. Hwang H. (2002). Generalized constrained canonical correlation analysis. Multivar. Behav. Res. 37, 163–195. 10.1207/S15327906MBR3702_01

[B107] Testa D. Jourde-Chiche N. Mancini J. Varriale P. Radoszycki L. Chiche L. (2021). Unsupervised clustering analysis of data from an online community to identify lupus patient profiles with regards to treatment preferences. Lupus 30, 1834–1837. 10.1177/09612033211033977 34313509

[B108] Tibshirani R. Guenther W. Trevor H. (2001). Estimating the number of clusters in a data set via the gap statistic. J. R. Stat. Soc. Ser. B 63, 411–423. 10.1111/1467-9868.00293

[B109] Torgerson W. S. (1952). Multidimensional scaling: I. Theory and method. Psychometrika 17, 401–419 10.1007/BF02288916 5217606

[B110] van de Velden M. D'Enza A. I. Palumbo F. C. (2017). Cluster correspondence analysis. Psychometrika. 82, 158–185. 10.1007/s11336-016-9514-0 27683298

[B111] Van der Maaten L. Hinton G. (2008). Visualizing data using t-SNE. J. Mach. Learn. Res. 9, 2579–2605.

[B112] Vellido A. (2020). The importance of interpretability and visualization in machine learning for applications in medicine and health care. Neural Comput. Appl. 32, 18069–18083. 10.1007/s00521-019-04051-w

[B113] Wang W. Yang J. Muntz R. (1997). “STING: A statistical information grid approach to spatial data mining,” in Vldb, Vol. 97, 186–195. Available online at: http://fmdb.cs.ucla.edu/Treports/970006.pdf

[B114] Warwick K. M. Lebart L. Morineau A. (1989). Multivariate descriptive statistical analysis (correspondence analysis and related techniques for large matrices). Appl. Stochast. Models Data Anal. 5, 175–175. 10.1002/asm.3150050207

[B115] Williams B. Mancia G. Spiering W. Rosei E. A. Azizi M. Burnier M.. (2018). ESC/ESH guidelines for the management of arterial hypertension: The task force for the management of arterial hypertension of the European Society of Cardiology and the European Society of Hypertension. J. Hypertens. 36, 1953–2041. 10.1097/HJH.0000000000001940 30234752

[B116] Windgassen S. Rona M-M. Kimberley G. Trudie C. (2018). The importance of cluster analysis for enhancing clinical practice: an example from irritable bowel syndrome. J. Mental Health 27, 94–96. 10.1080/09638237.2018.1437615 29447026

[B117] Wiwie C. Jan B. Richard R. (2015). Comparing the performance of biomedical clustering methods. Nat. Methods 12, 1033–1038. 10.1038/nmeth.3583 26389570

[B118] Xu R. Donald C. W. (2010). Clustering algorithms in biomedical research: a review. IEEE Rev. Biomed. Eng. 3, 120–154. 10.1109/RBME.2010.2083647 22275205

[B119] Yang J. Susanto R. Pasi F. (2021). Mean-shift outlier detection and filtering. Pattern Recognit. 115, 107874. 10.1016/j.patcog.2021.107874

[B120] Zhang T. Raghu R. Miron L. (1996). BIRCH: an efficient data clustering method for very large databases. ACM SIGMOD Rec. 25, 103–114. 10.1145/235968.233324

[B121] Zhao Y. Karypis G. (2002). Comparison of Agglomerative and Partitional Document Clustering Algorithms. Section: Technical Reports. Minnesota Univ Minneapolis Dept of Computer Science. Available online at: https://apps.dtic.mil/sti/citations/ADA439503 (accessed September 9, 2021).

[B122] Zhou F. L. Hirotaka W. Yuki T. Mathilde B. Dian K. Cyril E.. (2019). Identification of subgroups of patients with type 2 diabetes with differences in renal function preservation, comparing patients receiving sodium-glucose co-transporter-2 inhibitors with those receiving dipeptidyl peptidase-4 inhibitors, using a supervised machine-learning algorithm (PROFILE study): a retrospective analysis of a Japanese commercial medical database. Diabetes Obes. Metab. 21, 1925–1934. 10.1111/dom.13753 31050099 PMC6771907

[B123] Zwick W. R. Wayne F. V. (1986). Comparison of five rules for determining the number of components to retain. Psychol. Bull. 99, 432–442. 10.1037/0033-2909.99.3.432

