# Supplementary figures and images for: Qluster: An easy-to-implement generic workflow for robust clustering of health data
Source: Front Artif Intell. 2023 Feb 6;5:1055294. doi: 10.3389/frai.2022.1055294 (PMC9939832; doi:10.3389/frai.2022.1055294)

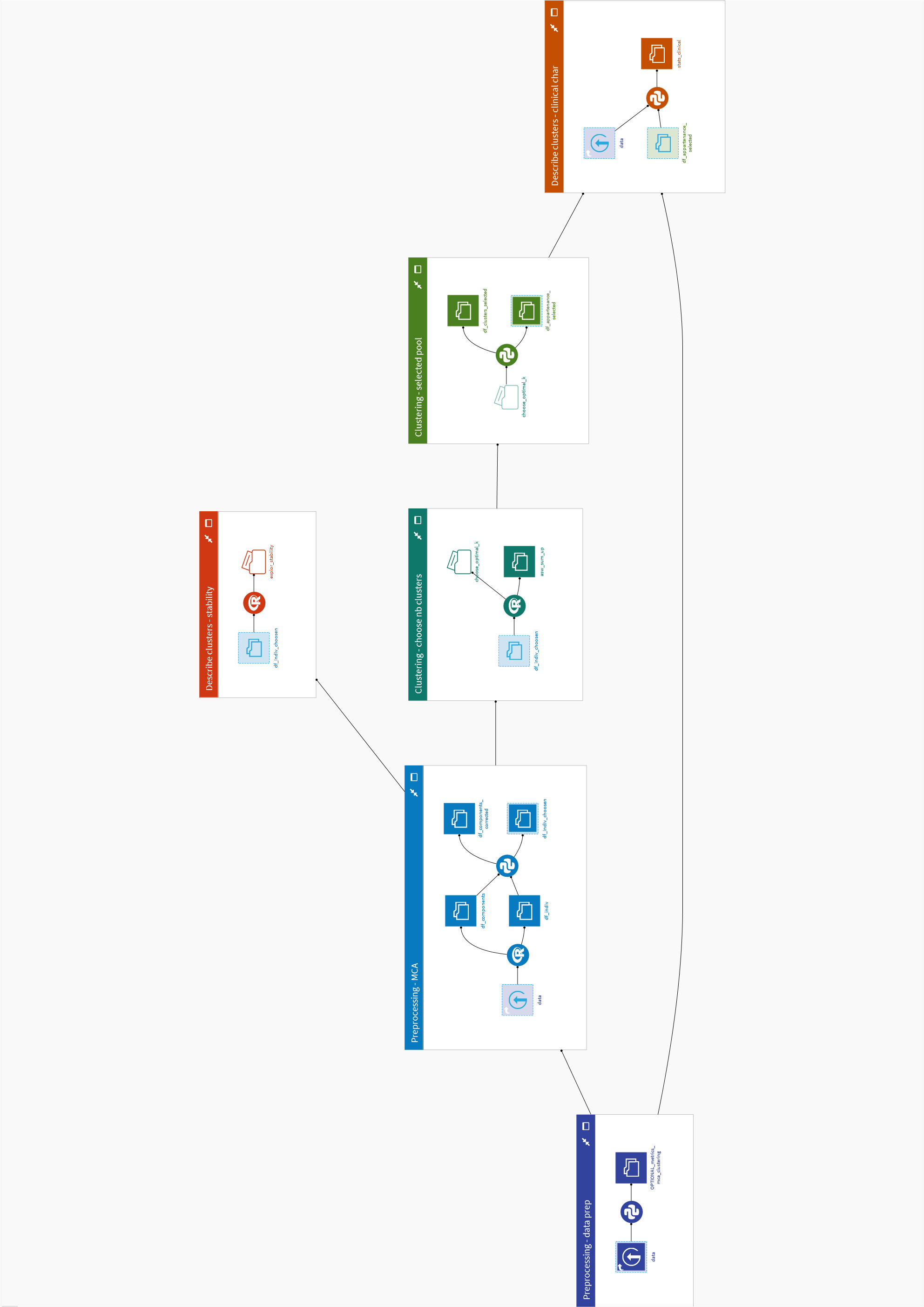

Supplement: Appendix B — Example of an implementation of the Qluster workflow on the Dataiku platform. [file Image_1.TIF]

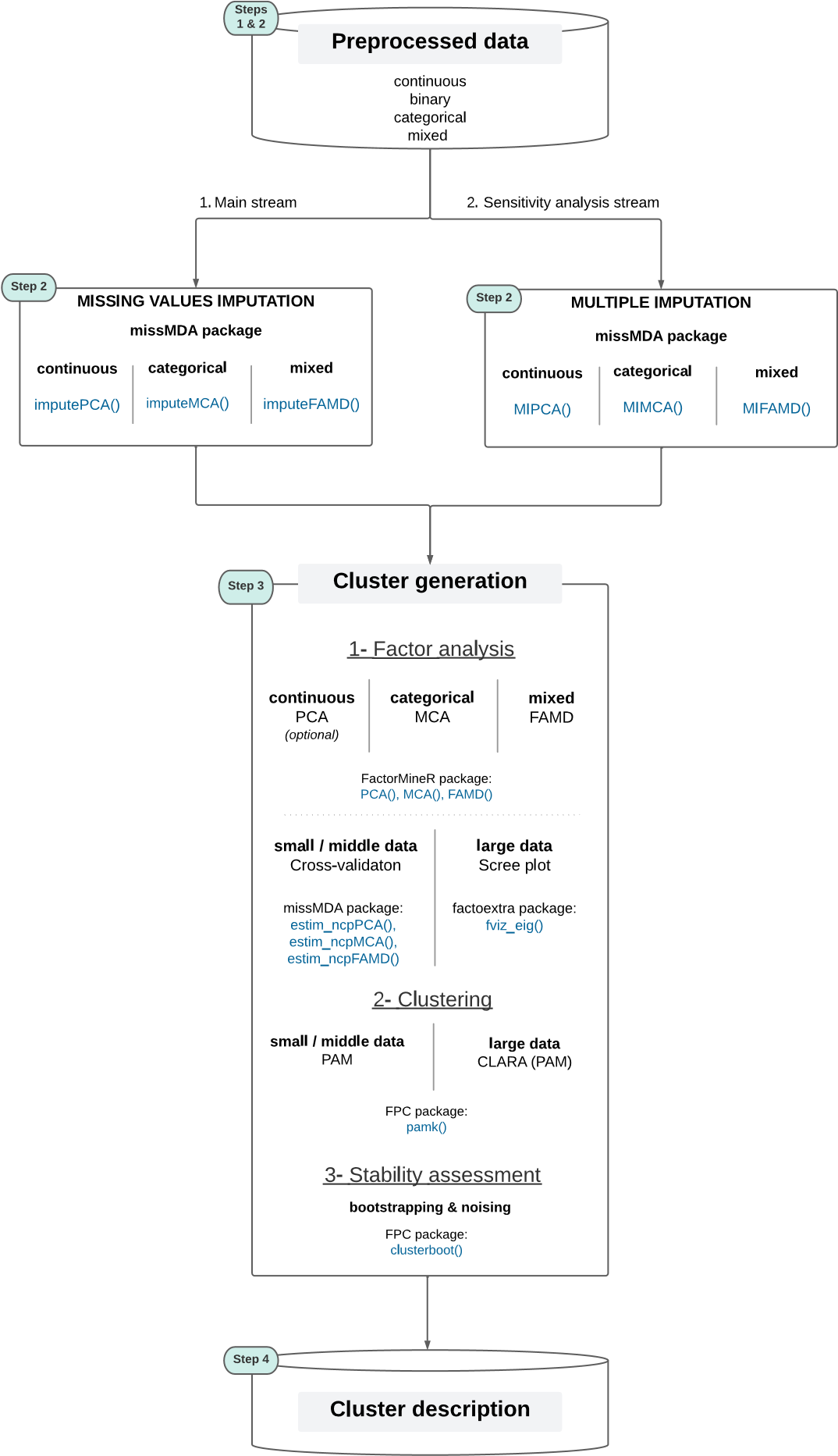

Supplement: Appendix E — Example of the Qluster workflow adapted for handling missing values. [file Image_2.TIF]
