# Supplementary material for: Qluster: An easy-to-implement generic workflow for robust clustering of health data
Source: Front Artif Intell. 2023 Feb 6;5:1055294. doi: 10.3389/frai.2022.1055294 (PMC9939832; doi:10.3389/frai.2022.1055294)
Supplement: Appendix C — R Code to implement Benzecri correction from MCA eigenvalues. [file Data_Sheet_2.docx]

## Appendix C. R Code to implement Benzecri correction from MCA eigenvalues

| correction_benz = function(eig, K){  *###*  *#Function to correct MCA eigenvalues using Benzecri correction  #Arguments:*  *# eig: vector of eigenvalues from MCA()*  *# K: number of qualitative variables (binary or categorical)*  *#Returns: eigenvalues and variances corrected  ###*  *# Benzecri correction*  eig_benz <- rep(0, length(eig))  selection <- eig > 1/K  eig_benz[selection] <- ((K/(K-1))*(eig[selection] - 1/K))^2  *# Save results# save results*  *var_benz <- 100*eig_benz/sum(eig_benz)*  *df_mca_benz <- cbind(eig_benz, var_benz, cumsum(var_benz))*  *colnames(df_mca_benz) = c("eig_benz", "%_var_benz", "cum_%_var_benz")*    *return(df_mca_benz)*  } |
| --- |
