# Supplementary material for: Qluster: An easy-to-implement generic workflow for robust clustering of health data
Source: Front Artif Intell. 2023 Feb 6;5:1055294. doi: 10.3389/frai.2022.1055294 (PMC9939832; doi:10.3389/frai.2022.1055294)
Supplement: Appendix D — Eigenvalues and variances explained with and without Benzecri correction. [file Data_Sheet_3.docx]

## Appendix D. Eigenvalues and variances explained with and without Benzecri correction

| **Dimension** | **Eigenvalue** | **Variance explained (in %)** | **Cumulative variances explained (in %)** | **Eigenvalue corrected by**  **Benzecri** | **Variances explained corrected by**  **Benzecri (in %)** | **Cumulative variances explained corrected by Benzecri (in %)** |
| --- | --- | --- | --- | --- | --- | --- |
| 1 | 0*.*29 | 23*.*6 | 23*.*6 | 0*.*04 | 92*.*1 | 92*.*1 |
| 2 | 0*.*16 | 13*.*3 | 36*.*9 | *<* 0*.*01 | 7*.*7 | 99*.*7 |
| 3 | 0*.*12 | 9*.*8 | 46*.*7 | *<* 0*.*01 | 0*.*2 | 99*.*9 |
| 4 | 0*.*12 | 9*.*5 | 56*.*1 | *<* 0*.*01 | 0*.*1 | 100*.*0 |
| 5 | 0*.*11 | 9*.*0 | 65*.*2 | − | − | 100*.*0 |
| 6 | 0*.*11 | 8*.*8 | 74*.*0 | − | − | 100*.*0 |
| 7 | 0*.*10 | 8*.*4 | 82*.*4 | − | − | 100*.*0 |
| 8 | 0*.*09 | 7*.*6 | 90*.*0 | − | − | 100*.*0 |
| 9 | 0*.*07 | 5*.*6 | 95*.*6 | − | − | 100*.*0 |
| 10 | 0*.*04 | 3*.*5 | 99*.*0 | − | − | 100*.*0 |
| 11 | 0*.*01 | 1*.*0 | 100*.*0 | − | − | 100*.*0 |
